# Associations of maternal night shift work during pregnancy with DNA methylation in offspring: a meta-analysis in the PACE consortium

**DOI:** 10.1186/s13148-024-01810-y

**Published:** 2025-01-22

**Authors:** Irene F. Marques, Carola Domènech-Panicello, Madelon L. Geurtsen, Thanh T. Hoang, Rebecca Richmond, Kristen Polinski, Lea Sirignano, Christian M. Page, Anne-Claire Binter, Todd Everson, Amber Burt, Michael Deuschle, Maria Gilles, Fabian Streit, Sunni L. Mumford, Per Magnus, Irwin K. M. Reiss, Marijn J. Vermeulen, Stephanie H. Witt, Inês Chaves, Edwina Yeung, Stephanie J. London, Mònica Guxens, Janine F. Felix

**Affiliations:** 1 Generation R Study Group, Erasmus MC, University Medical Center Rotterdam, Rotterdam, the Netherlands; 2 Department of Pediatrics, Erasmus MC, University Medical Center Rotterdam, Rotterdam, the Netherlands; 3 ISGlobal, Barcelona, Spain; 4 Universitat Pompeu Fabra (UPF), Barcelona, Spain; 5 CIBER Epidemiología y Salud Pública (CIBERESP), Instituto de Salud Carlos III, Madrid, Spain; 6 Division of Intramural Research, National Institute of Environmental Health Sciences, National Institutes of Health, Research Triangle Park, NC USA; 7 Department of Pediatrics, Division of Hematology-Oncology, Baylor College of Medicine, Houston, TX USA; 8 Dan L. Duncan Comprehensive Cancer Center, Baylor College of Medicine, Houston, TX USA; 9 Cancer and Hematology Center, Texas Children’s Hospital, Houston, TX USA; 10 Medical Research Council Integrative Epidemiology Unit, University of Bristol, Bristol, UK; 11 Population Health Sciences, Bristol Medical School, University of Bristol, Bristol, UK; 12 NIHR Oxford Health Biomedical Research Centre, University of Oxford, Oxford, UK; 13 Division of Population Health Research, Division of Intramural Research, Eunice Kennedy Shriver National Institute of Child Health and Human Development, Bethesda, MD USA; 14 Department of Genetic Epidemiology in Psychiatry, Medical Faculty Mannheim, Central Institute of Mental Health, Heidelberg University, Mannheim, Germany; 15 Centre for Fertility and Health, Norwegian Institute of Public Health, Oslo, Norway; 16 Department of Physical Health and Aging, Division for Physical and Mental Health, Norwegian Institute of Public Health, Oslo, Norway; 17 Gangarosa Department of Environmental Health, Rollins School of Public Health, Emory University, Atlanta, GA USA; 18 Department of Psychiatry and Psychotherapy, Medical Faculty Mannheim, Central Institute of Mental Health, Heidelberg University, Mannheim, Germany; 19 Medical Faculty Mannheim, Hector Institute for Artificial Intelligence in Psychiatry, Central Institute of Mental Health, Heidelberg University, Mannheim, Germany; 20 Department of Biostatistics, Epidemiology and Informatics and Department of Obstetrics and Gynecology, Perelman School of Medicine, University of Pennsylvania, Philadelphia, PA USA; 21 Department of Neonatal and Pediatric Intensive Care, Erasmus MC, University Medical Center Rotterdam, Rotterdam, the Netherlands; 22 Department of Molecular Genetics, Erasmus MC Cancer Institute, Erasmus MC, University Medical Center Rotterdam, Rotterdam, the Netherlands; 23 Department of Child and Adolescent Psychiatry/Psychology, Erasmus MC, University Medical Center Rotterdam, Rotterdam, the Netherlands; 24 ICREA, Barcelona, Spain

**Keywords:** Night shift work, Pregnancy, Cohort study, DNA methylation, Epigenetics

## Abstract

**Background:**

Night shift work during pregnancy has been associated with differential DNA methylation in placental tissue, but no studies have explored this association in cord blood. We aimed to examine associations of maternal night shift work with cord blood DNA methylation.

**Methods:**

A total of 4487 mother–newborn pairs from 7 studies were included. Maternal night shift work during pregnancy was ascertained via questionnaires and harmonized into “any” *versus* “no”. DNA methylation was measured in cord blood using the Illumina Infinium Methylation arrays. Robust linear regression models adjusted for relevant confounders were run in the individual cohorts, and results were meta-analyzed.

**Results:**

Maternal night shift work during pregnancy ranged from 3.4% to 26.3%. Three CpGs were differentially methylated in relation to maternal night shift work during pregnancy at a false discovery rate adjusted *P* < 0.05: cg10945885 (estimate (*β*) 0.38%, standard error (SE) 0.07), cg00773359 (*β* 0.25%, SE 0.05), and cg21836426 (*β* − 0.29%, SE 0.05). Associations of the identified CpGs were found in previous literature for gestational age and childhood and adolescent BMI. In a mouse model of prenatal jet lag exposure, information on offspring DNA methylation of ten homologous genes annotated to the 16 CpGs with *P* < 1 × 10^−5^ in our analysis was available, of which eight were associated (enrichment *P*: 1.62 × 10^−11^).

**Conclusion:**

Maternal night shift work during pregnancy was associated with newborn DNA methylation at 3 CpGs. Top findings overlapped with those in a mouse model of gestational jet lag. This work strengthens evidence that DNA methylation could be a marker or mediator of impacts of circadian rhythm disturbances.

**Supplementary Information:**

The online version contains supplementary material available at 10.1186/s13148-024-01810-y.

## Background

As an adaptation to the environment created by the earth’s rotation, many mammalian behaviors and physiological processes reflect a near 24-h circadian rhythm, from Latin “circa diem”, about a day [1, 2]. Daylight is the most important synchronizing agent of the circadian system, which is composed of a central clock in the brain and peripheral clocks in all other tissues [2]. Circadian rhythm disturbances, such as those encountered during jet lag or night shift work, are associated with adverse health outcomes in adults [3]. For instance, extended periods of night shift work among nurses are associated with increased risk of developing breast cancer [4] and type 2 diabetes [5]. According to Eurofound, the prevalence of shift work in Europe was 19% in 2015 [6]. In 2021, 38% of the general working population reported working for at least 2 h between 10 pm and 5am at least occasionally, including 27% among women [7].

Women exposed to shift work during pregnancy have an increased risk of pregnancy-related complications, including miscarriage, prematurity, and low birthweight offspring [8–10]. This warrants the study of circadian rhythm disturbances during pregnancy and its long-term implications for offspring health.

Differential DNA methylation has emerged as a potential mechanism underlying the associations of exposures during pregnancy with offspring health outcomes [11]. Prenatal exposures such as maternal smoking, body mass index (BMI), and folate levels during pregnancy have been robustly associated with differential DNA methylation in offspring [12–14]. Several animal models have been developed to study the long-term consequences of circadian rhythm disruption during pregnancy on offspring health [15]. Exposure of pregnant mice to circadian rhythm disturbances predisposes adult offspring to impaired cardiac and metabolic function and reduced bone mass [16–18]. Analysis of DNA methylation in adult offspring liver tissue indicated differential DNA methylation between groups exposed and unexposed to circadian rhythm disturbances [16]. In humans, exposure to maternal night shifts during pregnancy has been associated with differential DNA methylation in placental tissue at 57 CpGs at *P*_FDR_ < 0.05. In 53 out of 57 associations, exposure to night shifts was associated with lower DNA methylation [19]. As DNA methylation is tissue-specific, associations of night shift exposure in placental tissue may reflect different biological adaptations to circadian disruption than in other tissues. No studies have yet explored this association in offspring cord blood. Therefore, we examined associations of maternal night shift work during pregnancy with offspring cord blood genome-wide DNA methylation in a multi-cohort setting.

## Methods

### Participants

Seven studies collaborating in the pregnancy and childhood epigenetics (PACE) Consortium [20] participated in this study: the Avon Longitudinal Study of Parents and Children (ALSPAC) from the UK [21, 22], the Effects of aspirin in gestation and reproduction (EAGeR) randomized clinical trial from the USA [23], the Generation R Study from the Netherlands [24], the INfancia y Medio Ambiente (INMA) Project from Spain [25], the Norwegian Mother, Father and Child Cohort Study (two subcohorts: MoBa1 and MoBa2) [26–28], and the Pre-, Peri-, and Postnatal Stress: epigenetic impact on depression (POSEIDON) study from Germany [29, 30]. We excluded all twins and for non-twin siblings we included one sibling per mother, based on completeness of data or, if equal, randomly. Complete case analyses were performed at the cohort level. Further details can be found in Additional File 1. Informed consent was obtained for all participants, and all studies were approved by their local ethics committees.

### Maternal night shift work (exposure)

Maternal night shift work during pregnancy was ascertained independently by each study through questionnaires administered to the mothers at different time points. As such, each study had different definitions, e.g., based on frequency of night shift work or on number of hours worked in different time intervals. In ALSPAC, mothers were questioned about their current work life at both 18 and 32 weeks of gestation. In EAGeR, participants were asked about their current working schedule 2 months prior to pregnancy. In MoBa, pregnant women answered the questionnaire between the 15th and 25th week of pregnancy, and the question referred to current working conditions. In the Generation R Study, the questionnaire was received after the 25th week of gestation, and the question referred to the previous 3 months. In INMA, the questionnaire was received at 32 weeks and the questions referred to the full pregnancy. In POSEIDON, mothers received questionnaires at recruitment (3rd trimester), while the question referred to early-pregnancy working conditions. For the purpose of this study, the participating studies were requested to harmonize night shift work into "any" *versus* "no" night shift work exposure. Please see Additional File 1 for detailed exposure information.

### DNA methylation (outcome)

Epigenome-wide DNA methylation was measured in cord blood using the Illumina Infinium® HumanMethylation450 (6 cohorts) or EPIC BeadChip assays (1 cohort) (Illumina, San Diego, CA, USA). Quality control and normalization were conducted independently by each study using their preferred methods (see Additional File 1 for detailed methods). Untransformed autosomal beta values, ranging from 0 to 1, were used as outcome. Extreme DNA methylation values were winsorized for 2% of the participants per probe, 1% at the upper end and 1% at the lower end [31].

### Covariates

Included covariates were maternal covariates, cell types, batch, and child sex. Maternal covariates were maternal age (continuous, years), education (categorized according to study definition into two to four levels), and smoking status during pregnancy (preferably categorized into sustained smoking *versus* no smoking or quitting in first trimester; if that was not possible, then according to study definition). Proportions of seven blood cell subtypes (CD8 + Tcells, CD4 + Tcells, natural killer cells, B cells, monocytes, granulocytes, and nucleated red blood cells) were estimated based on DNA methylation using a cord blood-specific reference panel [32]. Batch effects were corrected using cohort-preferred methods, for example by including a batch variable in the model or by surrogate variable analysis with the sva R package [33]. As sample sizes for groups of non-European ancestry in the participating cohorts were not sufficiently large, cohort-specific analyses were restricted to participants of European ancestry. Please see Additional File 1 for details.

### Cohort-specific epigenome-wide association analyses

Each cohort analyst followed a pre-specified analysis plan and analytic code [11]. Robust linear regression models (rlm) were used for all cohort-specific epigenome-wide analyses. The basic (“crude”) model was adjusted only for child sex and batch. The main analysis was additionally adjusted for the maternal covariates and cell type proportions. A reduced main model, run as a sensitivity analysis, included all main model variables, except for cell type proportions. A link to the analytic code used in this project can be found in the **Availability of data and materials** section.

### Meta-analyses

We performed a two-stage meta-analysis [11], in which the results files from the cohort-specific epigenome-wide association studies were shared with the leading team and meta-analyses were run centrally at Erasmus MC in Rotterdam (NL). The PACE consortium employs rigorous quality control procedures for each meta-analysis [11]. We performed quality control of the individual cohort results with the QCEWAS R package [34], which checks for impossible effect sizes and *P* values and generates several plots (i.e., QQ plots with lambda (*λ*) values, volcano and Manhattan plots, and histograms of effect sizes and standard errors). After quality control, we ran fixed-effects inverse variance weighted meta-analyses in METAL [35]. Independent shadow meta-analyses were performed at ISGlobal in Barcelona (Spain), using the EASIER R package [36] for the quality control and GWAMA [37] for the meta-analyses. The results were compared and found to be identical. Additionally, cohort-specific epigenome-wide studies passed all the QC steps. We found some evidence of inflation at the cohort level, but most *λ* values were close to 1 (minimum *λ* = 0.96 and maximum = 1.61). As only one cohort used the EPIC array to assess DNA methylation, we only included probes from that cohort which overlapped with the 450 K array. Overall, probes measured in only one cohort (*N* = 383,683), probes mapped to the X and Y chromosomes (*N* = 10,232), and cross-reactive probes (*N* = 10,232) [38, 39] were excluded, leaving 429,959 CpGs in the main model meta-analysis. Probes listed as potentially polymorphic were flagged, but not excluded [38, 39]. We corrected for multiple testing individually for each meta-analysis model based on a 5% false discovery rate (FDR) [40]. Manhattan and volcano plots were created to visually present results. The *λ* value for the main meta-analysis model was 1.24. For the CpGs with a *P*_FDR_ value < 0.05, we created forest plots and calculated I-squared values using the EASIER R package, to assess heterogeneity across studies. To evaluate whether any individual study was a major driver of these FDR-significant findings, we conducted leave-one-out analyses, in which we repeated the meta-analysis leaving each one of the seven studies out at a time. All statistical analyses were performed in R [41], unless otherwise specified.

The full epigenome-wide meta-analyses results of the crude, main, and reduced main models can be found in Additional File 2 and in the Zenodo repository (link can be found in the **Availability of data and materials** section).

### Follow-up analyses

To examine potential functionality of the identified CpGs in a broader context, we performed four types of follow-up analyses: enrichment analyses, comparison with previous EWAS literature, examination of mQTLs and eQTMs, and examination of annotated genes in a mouse model of prenatal jet lag.

#### Enrichment analyses

We ran functional enrichment analyses using Gene Ontology (GO) and Kyoto Encyclopedia of Genes and Genomes (KEGG) in the R package MissMethyl [42], and we examined enrichment for tissue-specific regulatory components in eFORGE 2.0 [43] using an expanded set of 118 CpGs with unadjusted *P* values below 1 × 10^–4^, to assure sufficient input for these analyses. We considered results of these analyses to be significant if FDR-adjusted *P* values were < 0.05.

#### Comparison to previous EWAS literature

We looked up the FDR-significant CpGs in the EWAS Catalog [44], to examine whether these CpGs had previously been associated with other phenotypes. We also examined the effect estimates of the FDR-significant CpGs identified in the current study in previous EWASs of child phenotypes that are specifically relevant to our exposure of interest and its adverse health consequences: birthweight [45], gestational age [46], and BMI [47], as well as in the previous EWAS of maternal night shift work during pregnancy conducted in placental tissue [19]. Additionally, we examined the effect estimates of our FDR-significant CpGs in a recent EWAS of shift work in adults [48] as well as in an EWAS of sleep patterns in children [49]. Finally, we also examine the effect estimates from the hits reported in the placental EWAS of maternal shiftwork in our main model results.

When reporting the results of these comparative analyses, we report CpGs with unadjusted *P* values < 0.05 from the original studies as suggestive associations. Although DNA methylation at these CpGs was not significantly associated in the earlier studies, we consider it important to report them, as their nominal significance can be interpreted as supportive of our findings and may indicate potentially relevant biological mechanisms.

#### Examination of mQTLs and eQTMs

To examine whether there might be any genetic effects on the FDR-significant CpGs, we investigated whether these were associated with genetic variants, i.e., methylation quantitative trait loci (mQTLs), based on two available databases [50, 51]. The dataset published by Gaunt et al. is based on 1,018 mother–child pairs from the ARIES study to examine genetic influences on DNA methylation at different childhood stages and during pregnancy [50]. This dataset is smaller, but it can discern mQTLs specific to early life. Min et al. used a much larger dataset including 27,750 participants across all life stages in the GoDMC consortium to identify mQTLs and is therefore more powered, but not specific in terms of life stage [51]. We investigated the DNA methylation distribution for the FDR-significant CpGs and tested unimodality with the diptest R Package [52].

Similarly, we also examined the associations of the FDR-significant CpGs with gene expression in childhood blood, based on expression quantitative trait methylation (eQTMs) reported by the Human Early Life Exposome (HELIX) project [53]. This dataset includes blood autosomal *cis*-eQTMs from 832 children, adjusted for cell type proportions.

#### Examination of annotated genes in a mouse model of prenatal jet lag

To examine effects of sustained circadian disruption throughout pregnancy under controlled circumstances, we used the results of an existing mouse study, in which pregnant mice were subjected to a jet lag protocol. DNA methylation was measured in the offspring, and differentially methylated regions (DMRs) in relation to jet lag exposure were previously analyzed [16]. We examined whether the genes annotated to the 16 CpGs with unadjusted *P* values below 1 × 10^–5^ from our main model were also implicated in the mouse model, i.e., whether a DMR associated with jet lag exposure was present in the corresponding mouse gene. For this examination, we use a less stringent *P* value cutoff below 1 × 10^–5^, to indicate suggestive findings that may still represent relevant biology.

To summarize the jet lag animal model study, mating was conducted on a regular 12:12-h light–dark cycle. Then, pregnant mice were assigned to one of the three groups: a 12:12-h light–dark cycle and a serial 8-h advanced or delayed light–dark cycles. After birth, all groups were exposed to a regular 12:12-h light–dark cycle and several examinations were performed on the offspring, including liver DNA methylation analysis in 4-week-old male mice. Differently methylated regions (DMRs) between control and the jet lag groups were examined with the MeD-seq method [54]. DMRs were classified as regions with more than 10 CpGs, more than 100 bp long, and with a more than twofold change between controls and at least one of the jet lag groups.

The lookup of mice DMRs was done at gene level, taking the nearest gene annotated to the human CpGs and then looking for DMRs in the correspondent mouse gene. For one CpG without annotation, we identified the closest upstream and downstream genes in the UCSC Genome Browser. One-tailed Fisher’s exact test was used to calculate enrichment for gene-level replication.

## Results

### Participants

A total of 4487 mother–child pairs from seven cohorts from the PACE Consortium were included in the main meta-analysis. Cohort-specific characteristics can be found in Table 1. The proportion of children exposed to maternal night shift work during pregnancy ranged from 3.4% to 26.3%, and the participants were of European ancestry only (Additional File 1).

**Table 1:** Cohort-specific descriptive statistics

	ALSPAC	EAGeR	Generation R	INMA	MoBa1	MoBa2	POSEIDON
Sample size (main model)	810	354	1162	339	946	583	293
Maternal shift work (yes, N (%))	79 (9.8)	93 (26.3)	43 (3.7)	15 (4.4)	202 (21.4)	119 (20.4)	28 (9.6)
Maternal age (years, mean (SD))	30.2 (4.3)	28.3 (4.4)	31.8 (4.1)	31.1 (4.0)	30.1 (4.2)	30.0 (4.4)	31.5 (4.9)
Maternal BMI (kg/m^2^, mean (SD))	22.9 (3.8)	25.2 (5.6)	24.1 (4.0)	23.5 (4.2)	24.0 (4.1)	24.2 (4.4)	24.6 (5.2)
Maternal education (N (%)) Low Medium High^a^ High^b^	408 (50.4) 241 (29.8) 161 (19.9) –	34 (9.6) – 320 (90.4) –	18 (1.5) 363 (31.2) 781 (67.2) –	83 (24.5) 143 (42.2) 113 (33.3) –	57 (6.0) 299 (31.6) 432 (45.7) 158 (16.7)	36 (6.2) 184 (31.6) 249 (42.7) 114 (19.6)	30 (10.2) 88 (30.0) 175 (59.7) –
Maternal smoking (N (%)) no/until pregnancy was known sustained smoking	740 (91.4) 70 (8.6)	328 (92.7) 26 (7.3)	1009 (86.8) 153 (13.2)	296 (87.3) 43 (12.7)	816 (86.3) 130 (13.7)	520 (89.2) 63 (10.8)	260 (88.7) 33 (11.3)
Child sex (female, N (%))	400 (49.4)	178 (50.3)	579 (49.8)	172 (50.4)	442 (46.7)	252 (43.2)	140 (47.8)
Birth weight (grams, mean (SD))	3492 (488)	3360 (468)	3560 (497)	3268 (418)	3638 (546)	3642 (541)	3411 (487)
Gestational age (weeks, mean (SD))	39.6 (1.5)	38.9 (1.5)	40.2 (1.4)	39.7 (1.4)	39.5 (1.6)	39.5 (1.6)	39.2 (1.3)
Preterm births (gestation < 37 weeks, N (%))	22 (2.7)	26 (7.3)	31 (2.7)	0 (0)	31 (3.3)	29 (5.0)	9 (3.0)

Values are presented as mean and standard deviation (SD) for continuous variables and counts (N) and percentage (%) for categorical variables

^a^High and ^b^High: only MoBa used two categories of higher education. High^a^ refers to less than 4 years of university and High^b^ to more than 4 years

### Meta-analyses

**Fig. 1:**
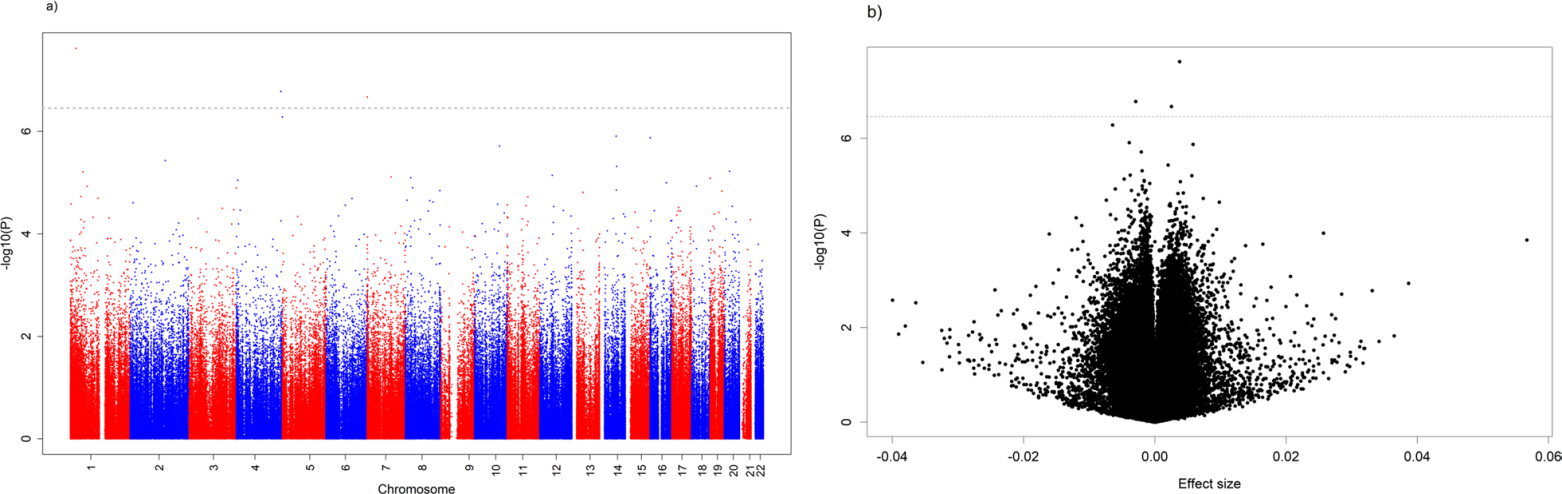
Manhattan (**a**) and volcano (**b**) plots of the main model meta-analysis results. Dashed lines indicate the cutoff for the *P* value adjusted for FDR at 5% significance level

**Fig. 2:**
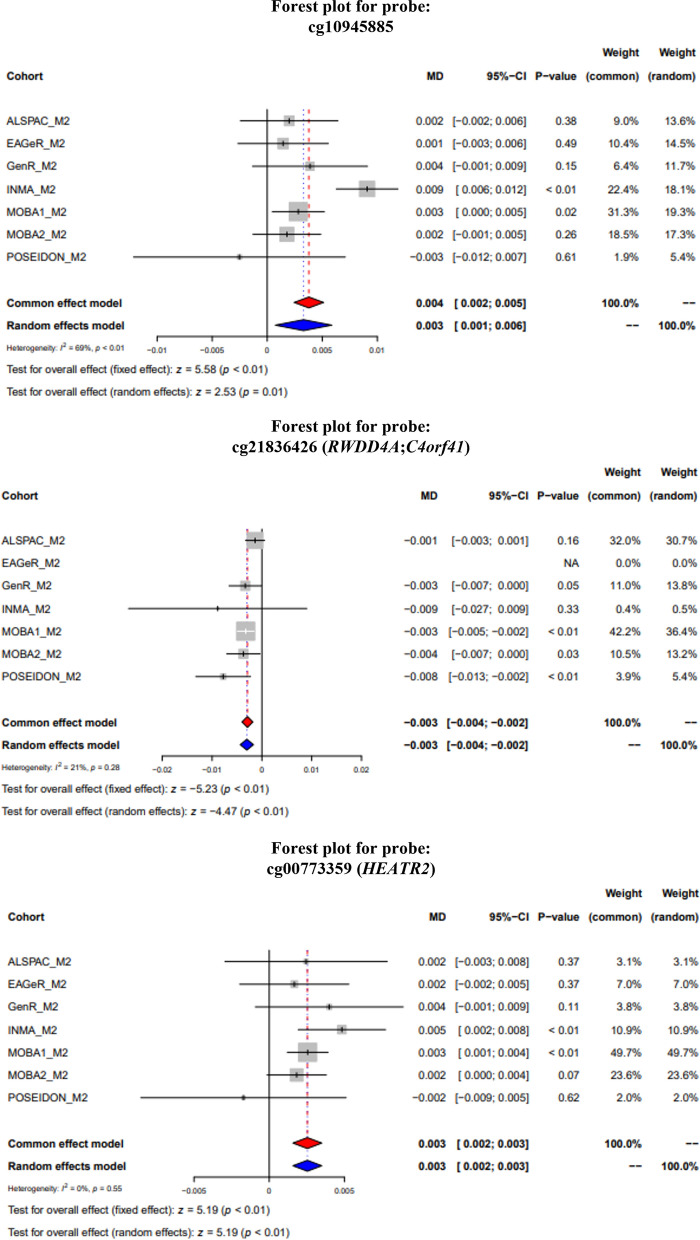
Forest plots for the FDR-significant CpGs main meta-analyses. *MD*: mean difference; 95%-*CI*: 95% confidence interval. For each study, the vertical line corresponds to the study effect estimate for that CpG; the horizontal line corresponds to the 95% CI. These lines are white when the 95% CI is completely inside the gray box. The gray box size corresponds to the study weight in the fixed-effects (or common effect) meta-analysis. Red and blue diamonds represent the results of the fixed-effects and random-effects meta-analyses, respectively. Dashed vertical red and blue lines are added to allow easier comparison of each study effect estimate with the fixed-effects and random-effects meta-analyses effect estimates, respectively. The main model was adjusted for child sex, batch, cell type proportions, maternal age, education, and smoking status during pregnancy. Cg21836426 is not part of the EPIC array, and hence, EAGeR does not have results for this CpG

In the main model, we identified three CpGs at *P*_FDR_ < 0.05 differentially methylated in relation to exposure to maternal night shift work during pregnancy, as compared to no exposure: cg10945885 (0.38% higher methylation, standard error (SE) = 0.07, *P* = 2.40 × 10^–8^), cg00773359 (0.25% higher methylation, SE = 0.05, *P* = 2.14 × 10^–7^), and cg21836426 (0.29% lower methylation, SE = 0.05, *P* = 1.67 × 10^–7^). Meta-analysis results for the 16 CpGs with a *P* value below 1 × 10^−5^ are shown in Table 2. A link to the full results for crude, main, and reduced main models can be found in the Availability of data and materials section. Only cg21836426 had *P*_FDR_ < 0.05 in all models. Manhattan and volcano plots for the main model are presented in Fig. 1 and for the crude and reduced main model in Additional File 3, Supplementary Fig. 1. QQ plots for all models are in Additional File 3, Supplementary Fig. 2. The Spearman genome-wide correlation of effect sizes between the main and reduced main models was 0.88 (Additional File 3, Supplementary Fig. 3). Forest plots and leave-one-out plots for the three significant CpGs can be found in Fig. 2 and in Additional File 3, Supplementary Figs. 4, respectively. The I^2^ values were 69, 21, and 0 for cg10945885, cg21836426, and cg00773359, respectively. We believe the high heterogeneity for cg10945885 is driven both by POSEIDON, which had an effect estimate in the opposite direction of the remaining cohorts, and also by INMA, which had a relatively large effect estimate and slightly higher than the remaining cohorts, although still in the same direction. For cg10945885, the CpG with highest heterogeneity, visual inspection of the forest and leave-one-out plots showed that the INMA cohort had a relatively strong effect on the meta-analysis result. However, results for the meta-analysis without INMA were still significant at *P* < 0.05, as seen in the leave-one-out plot. For cg21836426, leaving out the ALSPAC cohort slightly affected the meta-analysis result, but including ALSPAC gave a more conservative effect estimate. Cg00773359 showed the most stable results. Random effects meta-analyses showed similar results to the fixed-effects meta-analyses (Fig. 2).

**Table 2:** Main model meta-analysis results for the 16 CpG sites with *P* < 1 × 10^−5^

Marker name	Effect (β) ^a^	SE^a^	*P* value	Direction^b^	I^2^	Chr	Position	Gene Region	Relation to Island	Nearest Gene	FDR *P* value
cg10945885	0.38	0.07	2.40 × 10^–8^	+ + + + + + -	69	1	22,665,067	–	N_Shelf	–	0.01
cg21836426	− 0.29	0.06	1.67 × 10^–7^	- ? – – -	21	4	184,580,106	5'UTR 1stExon; TSS1500	Island	RWDD4A*; C4orf41*	0.03
cg00773359	0.25	0.05	2.14 × 10^–7^	+ + + + + + -	0	7	805,214	Body	N_Shelf	HEATR2*	0.03
cg25933594	− 0.64	0.13	5.24 × 10^–7^	– – – -	76	4	190,751,062	–	–		0.06
ch.14.955325R	− 0.39	0.08	1.24 × 10^–6^	– - ? – -	0	14	69,350,231	Body	–	*ACTN1*	0.10
cg01005536	0.58	0.12	1.35 × 10^–6^	+ + + — + + +	31	16	532,596	Body	Island	*RAB11FIP3*	0.10
cg08437570	− 0.21	0.04	1.95 × 10^–6^	– – – -	13	10	104,195,699	TSS1500	S_Shelf	*MIR146B*	0.12
cg09670616	0.20	0.04	3.70 × 10^–6^	+ ? + + — + +	79	2	145,277,790	Body 5'UTR 1stExon	N_Shelf	*ZEB2*	0.20
cg02311152	− 0.19	0.04	4.86 × 10^–6^	– + – –	0	14	71,276,396	TSS1500	Island	*MAP3K9*	0.23
cg23913995	− 0.38	0.08	6.00 × 10^–6^	- + – – -	14	20	21,490,665	–	Island		0.24
cg04079538	0.56	0.12	6.16 × 10^–6^	+ + + + + + +	0	1	51,811,558	TSS1500	S_Shore	*TTC39A*	0.24
cg05529152	− 0.47	0.10	7.27 × 10^–6^	+ – – –	42	12	52,414,843	–	N_Shelf		0.24
cg22087659	− 0.16	0.04	7.77 × 10^–6^	– + – –	0	7	99,516,845	1stExon	Island	*TRIM4*	0.24
cg23696886	− 0.16	0.04	8.09 × 10^–6^	– – – -	25	8	22,437,193	TSS1500 5'UTR	S_Shore	*PDLIM2*	0.24
cg01039573	0.39	0.09	8.26 × 10^–6^	+ + + + + + +	0	19	457,696	Body	N_Shore	*SHC2*	0.24
cg11973682	− 0.08	0.02	9.00 × 10^–6^	- ? – – -	32	4	5,890,633	Body TSS1500	Island	*CRMP1*	0.24

We report CpGs with unadjusted with *P* < 1 × 10^−5^, to indicate suggestive findings that may still represent relevant biologic mechanisms

^a^Effect size and SE are presented as % difference in DNA methylation for exposed as compared to unexposed groups

^b^Cohorts are ordered as follows: ALSPAC, EAGeR, Generation R, INMA, MoBa1, MoBa2, POSEIDON

*β*: effect estimate; *SE*: standard error; I^2^: heterogeneity; *Chr*: chromosome; *FDR*: false discovery rate; 5'*UTR*: 5ʹ untranslated region; TSS1500: 1500 bases upstream of transcriptional start site

Results presented correspond to main model EWAS for the associations of maternal shift work during pregnancy and newborn cord blood DNA methylation, adjusted for child sex, maternal educational, maternal age, maternal smoking, batch effects and cell type proportions

^*^Alternative gene names: *RWDD4A* = *RWDD4*; *C4orf41* = *TRAPPC11*; *HEATR2* = *DNAAF5*

### Follow-up analyses

#### Enrichment analyses

Functional enrichment analyses on the 118 CpGs with *P* < 1 × 10^−4^, a cutoff for suggestive association which gives a sufficient number of CpGs for meaningful analyses [47, 55–57], did not show FDR-significant pathways for either GO or KEGG (Additional File 3, Supplementary Table 1). Additionally, there was no significant enrichment for tissue-specific regulatory elements (Additional File 3, Supplementary Fig. 5).

#### Comparison to previous EWAS literature

A lookup of our three FDR-significant CpGs in the EWAS Catalog showed that DNA methylation at cg10945885, which was not annotated to a gene by the Illumina annotation, was associated with childhood age changes from birth until 18 years old in blood [58] and with differential DNA methylation in buccal cells *versus* whole blood in children [59]. DNA methylation at cg21836426, annotated to *RWDD4A* (5’UTR/ first exon) and *C4orf41* (alternative gene name *TRAPPC11*) (TSS1500), was previously associated with progressive supranuclear palsy, a form of tau-related dementia, in adults [60]. DNA methylation at cg00773359, annotated to the gene body of *HEATR2* (alternative gene name *DNAAF5*), was previously associated with childhood age changes from birth until 18 years old in blood [58], gestational age in fetal brain [61] and with acoustic cry variation in buccal cells from preterm infants [62].

The examination of our FDR-significant CpGs in previous EWASs of child phenotypes specifically relevant to maternal night shift work and its adverse health consequences revealed suggestive associations (unadjusted *P* < 0.05) for DNA methylation at cg00773359 in cord blood with gestational age [46] and with late-childhood BMI [47]. DNA methylation at cg10945885 and cg21836426 in whole blood was associated with adolescent BMI (Table 3). No associations were found in the longitudinal EWAS of sleep in children [49] (Table 3), and the three CpGs identified in our study were not found among the CpGs with *P* < 5 × 10^−4^ of the EWAS of shift work in adults [48].

**Table 3:** Associations of maternal shift-work-related CpG sites with relevant child outcomes in previous studies

Phenotype	cg10945885 ( +)			cg21836426 (-)			cg00773359 ( +)		
	Effect size	SE	*P* value	Effect size	SE	*P* value	Effect size	SE	*P* value
Birthweight (grams) **(45)**^**1**^	− 27.19	21.33	0.20	− 10.91	22.46	0.63	− 11.04	28.15	0.70
Gestational age (weeks) **(46)**^**1**^	7.76 × 10^–6^	2.77 × 10^–5^	0.78	− 2.24 × 10^–5^	2.30 × 10^–5^	0.33	− **4.02 × 10**^**–5**^	**1.87 × 10**^**–5**^	**0.03**
Child BMI, Model A (SDS) **(47)**^**1**^	0.002	0.95	0.99	0.98	1.12	0.38	1.38	1.27	0.28
Child BMI, Model B (SDS) **(47)**^**1**^	0.19	0.68	0.78	0.89	1.10	0.42	**1.63**	**0.84**	**0.05**
Child BMI, Model C (SDS) **(47)**^**1**^	− 0.72	0.94	0.45	0.63	1.40	0.65	0.31	1.38	0.82
Child BMI, Model D (SDS) **(47)**^**1**^	− **1.87**	**0.77**	**0.02**	− **2.27**	**1.07**	**0.03**	− 0.09	1.24	0.94
Parent-reported sleep duration in school age (SDS) **(49)**^**1**^	0.71	1.03	0.49	0.40	1.05	0.70	− 0.94	1.14	0.41

( +)/(-): direction of the association in current study

*SE* standard error, 1: numbers in brackets represent citations to the original EWAs; *SDS* standard deviation scores

Model A: longitudinal associations of cord blood DNA methylation with early-childhood BMI (2–5 years)

Model B: longitudinal associations of cord blood DNA methylation with late-childhood BMI (5–10 years)

Model C: cross-sectional associations of childhood blood DNA methylation with childhood BMI (2–10 years)

Model D: cross-sectional associations of adolescent blood DNA methylation with adolescent BMI (14–18 years)

Effect sizes, SE and *P* values copied directly from original studies. Please refer to each study for more details

Bolded values correspond to unadjusted *P* values < 0.05 in the original study

A prior EWAS on maternal circadian disruption during pregnancy in placental tissue identified 57 CpG at 5% FDR, 10 of which also met the Bonferroni significance threshold [19]. Of those, 51 were present in our data, of which five had an unadjusted *P* value < 0.05, but showed an opposite direction of effect in the placenta as compared to cord blood (Additional File 3, Supplementary Table 2). All nine available Bonferroni-significant CpGs had an opposite direction of effect between the studies, with only one, cg21373996, having an unadjusted *P* value < 0.05 in our cord blood analyses. Among the three CpGs we found to be significantly associated with shift work in cord blood, only cg00773359 was suggestively associated before multiple testing correction in placental tissue, again in the opposite direction (Table 4).

**Table 4:** Associations of maternal shift work with cord DNA methylation for placental Bonferroni CpGs and vice versa

CpG sites previously identified as differentially methylated in relation to maternal shift work in placental tissue				
		Effect size^1^	SE^1^	*P* value^1^
	cg14377596 (-)	0.0000	0.0100	0.72
	cg14168733 (-)	0.0700	0.2100	0.74
	cg01411786 (-)	0.0000	0.0200	0.86
	cg25342875 (-)	0.0500	0.1100	0.62
	cg06667732 (-)	0.1700	0.2700	0.52
	cg21373996 (-)	**0.1500**	**0.0700**	**0.03**
	cg14814323 (-)	0.1700	0.0900	0.06
	cg14858786 (-)	0.0400	0.0300	0.17
	cg08082763 (-)	0.1000	0.0900	0.25
CpG sites identified as differentially methylated in relation to maternal shift work in cord blood				
		Effect size^2^	SE^2^	*P* value^2^
	cg10945885 ( +)	− 0.0076	0.0082	0.35
	cg21836426 (-)	− 0.0019	0.0018	0.29
	cg00773359 ( +)	− **0.0025**	**0.0011**	**0.03**

1: Effect size, SE and *P* value correspond to the results of a lookup of these CpGs in the results of the current cord blood study

2: Effect size, SE and *P* value correspond to the results of a lookup of these CpGs in the results of the previously published EWAS of maternal shift work in placental tissue (19)

( +)/(-): direction of the association in the original study; SE: standard error

Bolded values: unadjusted *P* value < 0.05 in the lookup study

#### Examination of mQTLs and eQTMs

In the ARIES mQTL database [50], 16 *trans*-mQTL single nucleotide polymorphisms (SNPs) were associated with the three FDR-significant CpGs. Particularly, in cord blood, two SNPs, rs117371715 and rs183291851 (both with MAF = 0.01), were associated with methylation at cg21836426. In the Genetics of DNA Methylation Consortium (GoDMC) database [51], 9 *cis*-mQTLs were identified in association with cg10945885, but none with cg00773359 or cg21836426 (Additional File 3, Supplementary Tables 3 and 4). Density plots for the FDR-significant CpGs are shown in Additional File 3, Supplementary Figs. 6–12. The *P* values for the diptests were all > 0.05, indicating that we found no evidence for strong differential DNA methylation by genotype in our populations.

In the HELIX eQTM database [53], DNA methylation at cg00773359 was associated with expression of 4 transcripts, but none of the associations survived multiple testing correction (Additional File 3, Supplementary Table 5). DNA methylation at cg10945885 and cg21836426 was not associated with gene expression in *cis*.

#### Examination of annotated genes in a mouse model of prenatal jet lag

From the 18 human genes annotated to the top 16 CpGs (*P* < 1 × 10^−5^), 10 homologous genes were present in the mouse model and could therefore be examined. Of these, 8 genes contained a DMR in liver tissue of the jet lag animal model, which represents significant enrichment (Fisher’s *P* = 1.62 × 10^−11^, Additional File 3, Supplementary Table 6).

## Discussion

This meta-analysis of seven studies from the PACE Consortium, with a total of 4487 mother–child pairs, revealed some evidence for associations of maternal night shift work exposure with offspring DNA methylation. There were three CpGs associated at genome-wide significance: cg10945885 and cg00773359 were positively associated and cg21836426 was inversely associated with maternal night shift work exposure during pregnancy. Some associations of these three CpGs in previous EWASs of gestational age [45] and childhood BMI [47] as well as with gene expression [53] were found. There was a significant overlap with differentially methylated regions identified in liver in a mouse model of jet lag [16].

Exposure to shift work in adults has been associated with differential DNA methylation [48, 63]. In newborns exposed to maternal shift work during pregnancy, a particularly sensitive period of development, differential DNA methylation has only been explored in placental tissue [19]. In our study, we found differential DNA methylation in cord blood, a tissue that may reflect different biological adaptations to circadian disruption. The magnitude of the effect sizes found in our study is relatively small, which is in line with other EWASs on environmental exposures and DNA methylation [64]. It is thought that the accumulation of differential DNA methylation at many epigenome-wide sites, each with a very small effect, rather than at a single site with a large effect, may be involved in downstream biological processes.

Of the three genome-wide significant CpGs, cg00773359 is annotated to *HEATR2*. Mutations in this gene have been associated with primary ciliary dyskinesia [65]. Cg21836426 is annotated to both the *RWDD4A* and *C4orf41* genes. Mutations in *C4orf41* have been associated with myopathies, intellectual disability, and cerebral atrophy [66, 67]. *RWDD4A* has not been robustly associated with any phenotype in previous literature. Cg10945885 was not annotated to a gene in the Illumina annotation, but *ZBTB40* and *WNT4* are the closest genes, according to UCSC Genome Browser. Variants in *ZBTB40* have been associated with inflammatory bowel disease and low bone density [68] and *WNT4* with sex-determination developmental disorders [69, 70]. Overall, the potential biological mechanisms linking these genes to child health outcomes associated with maternal night shift work during pregnancy need to be further studied.

The examination of our FDR-significant CpGs in previous EWASs of phenotypes associated with maternal circadian rhythm disruption during pregnancy showed that cord blood methylation at cg00773359 was inversely associated with gestational age [46] and with late-childhood BMI [47], before multiple testing correction. DNA methylation at cg10945885, which had a positive association in our study, was negatively associated with cross-sectionally measured BMI in adolescence (unadjusted *P* value < 0.05) [47]. Maternal shift work during pregnancy has been associated with adverse birth outcomes, such as low birthweight, which, in turn, has been associated with higher childhood BMI [71]. Therefore, a negative association of cg10945885 with cross-sectional BMI in adolescence was not in line with the expected direction of effect. However, DNA methylation is known to change with age [58], so our findings at birth may not reflect biological processes in adolescence. Overall, our findings may support a potential role for DNA methylation as a mediator of the associations of early-life circadian rhythm disturbances with child health, but further studies are needed to disentangle the biological mechanisms.

Only one previous study in humans has examined the association of maternal night shift work during pregnancy with offspring DNA methylation, but this was done in placental tissue [19]. That study found differential DNA methylation at several CpGs, but none of the results were replicated in our study of cord blood. Cg00773359, which was positively associated with maternal night shift work in our study, was inversely associated with maternal night shift work in placental tissue. Likewise, the lookup of the placenta tissue hits in our study revealed effect sizes in opposite directions. Placental and cord blood DNA methylation reflect different and tissue-specific methylation patterns, which may explain the different findings. Of note, also for maternal smoking in pregnancy, where extensive differential DNA methylation has been identified, and for maternal pre-pregnancy BMI, overlap between findings for cord blood and placenta is minimal [72, 73].

A lookup of our findings in the childhood and pregnancy mQTL database [50] identified 16 *trans*-mQTLs for the FDR CpGs, two of which were specifically found at birth, for cg21836426. In the GoDMC database [51], we did not find mQTLs in association with cg21836426. This suggests that a potential genetic influence on DNA methylation at cg21836426 may be present at birth or in early life but not at later stages of life. We found some evidence for an association of cg00773359 with gene expression in childhood in the HELIX *cis*-eQTM database, but the associations did not survive multiple testing correction. We did not find *cis*-eQTM for the other two CpGs.

Furthermore, the lookup of the 118 CpGs with *P* < 1 × 10^−4^ in the GO, KEGG, and eFORGE bioinformatics tools did not reveal any indication for enriched biological (regulatory) processes. The number of CpGs used in the searches might be limiting the discovery of meaningful pathways. We refrained from further increasing the *P* value cutoff, which would increase the number of CpGs added to the pathway analysis, as the weaker associations with night shift work may introduce too much noise in the output.

DNA methylation at the genes annotated to the 16 CpGs with a *P* value < 1 × 10^−5^ had a high gene-level replication in the jet lag mouse model. The comparison of human *versus* mouse findings is not straightforward, and the methodologies used in the studies were different. Nevertheless, the stringent criteria to classify a DMR in the animal model limits the potential for false-positive findings. Additionally, DNA methylation in the mouse model was measured in liver tissue, a tissue that is potentially more relevant for the study of metabolic conditions, such as those associated with maternal night shift work, than cord blood. Although cross-tissue correlations of DNA methylation levels between blood and liver in humans have been reported to be generally low [74], the consistency of the findings between our human blood and mouse liver findings adds to the evidence for associations of circadian rhythm disturbance with DNA methylation at these loci.

Our study had several limitations. First, information on maternal night shift work during pregnancy was self-reported and collected using questionnaires at varying times prior to or during pregnancy, with the question pertaining to varying time windows and having different response options. The percentages of exposure to night shift work varied between the cohorts. This is likely related to the timing of filling out the questionnaire, with questionnaires earlier in pregnancy giving higher percentages, while the questionnaires in later pregnancy correspond to the lower exposure percentage. Only one study, EAGeR, assessed night shift work in the preconception period. We believe it is likely that the working conditions and shiftwork pattern continue at least from preconception into early pregnancy. Moreover, the leave-one-out meta-analysis of the FDR-hits did not reveal significant differences when EAGeR participants were left out. Therefore, the earlier collection time point did not seem to influence the meta-analysis result unduly. Second, the different timing and varying response options led to a relatively coarse classification of the exposure into “any” *versus* “no” night shift work during pregnancy. This may have limited our ability to find associations. Future studies with more detailed information on maternal working conditions during the periconceptional period and in pregnancy are needed to shed further light on the intricacies of timing- and dose-effects of exposure to night shift work in pregnancy. Third, the Illumina Infinium Human Methylation450 and EPIC BeadChips cover only 2–3% of all CpG sites in the DNA, and thus, DNA methylation at other unmeasured CpG sites may also be associated with exposure to night shift work. Fourth, we examined DNA methylation in cord blood, which may not represent the main target tissue in relation to future health outcomes. Fifth, the participants in the current study are all from European ancestry, so generalizability to other ancestries requires further study. Last, even though we adjusted our analyses for several confounders, residual confounding due to unmeasured factors might still be present, especially concerning occupational exposures, for example the risk of exposure to specific chemicals in industrial or cleaning jobs.

Regarding the study strengths, this is the first meta-analysis of epigenome-wide association studies examining the associations of maternal night shift work during pregnancy with offspring cord blood DNA methylation in a large sample of mothers and children from multiple countries. The studies included in this meta-analysis followed a predefined analysis plan and analytic code, which is standard practice in the PACE Consortium [20], and limits potential variation between cohorts and errors in the analyses.

Our results support further research into DNA methylation as a potential molecular mechanism underlying the associations of maternal night shift work during pregnancy with offspring health outcomes. Studies with more detailed information on maternal working conditions during pregnancy are still needed. Wearable devices to register sleep and wake patterns have become widely available in recent years and could provide a more detailed assessment of circadian rhythm disruptions in future studies.

## Conclusion

Maternal night shift work during pregnancy was associated with newborn DNA methylation at three CpGs, with potential associations with child gestational age and BMI. Top findings overlapped with those in a mouse model of jetlag, a related exposure. This work strengthens evidence that DNA methylation could be a marker or mediator of impacts of circadian rhythm disturbances.

## Supplementary Information

Additional file1 (DOCX 98 KB)

Additional file2 (XLSX 232625 KB)

Additional file3 (DOCX 2782 KB)

## Data Availability

Access to individual cohort-level data can be requested through the principal investigators of the individual studies and may be subject to local, national and international rules and regulations. Information on the study cohorts that contributed is available in Additional File 1: Cohort-specific Methods. The analytical code used for the analyses conducted in this article is available on GitHub, accessible through this link: https://github.com/i-marques/EWAS_shift_work_cordblood_2024. The datasets generated during the current study (genome-wide meta-analysis summary results) are available in the Zenodo repository (10.5281/zenodo.13143037), accessible via this link: https://zenodo.org/records/13143037.

## Abbreviations

ALSPAC: Avon Longitudinal Study of Parents and Children
BMI: Body mass index
bp: Base pairs
CpG: Cytosine–guanine dinucleotide site
DMR: Differentially methylated region
DNA: Deoxyribonucleic acid
EAGeR: Effects of aspirin in gestation and reproduction
eQTM: Expression quantitative trait methylation
EWAS: Epigenome-wide association study
FDR: False discovery rate
INMA: INfancia y medio ambiente
MoBa: Norwegian Mother, Father and Child Cohort Study
mQTL: DNA methylation quantitative trait locus
PACE: Pregnancy And Childhood Epigenetics Consortium
POSEIDON: Pre-, Peri-, and postnatal stress: epigenetic impact on depression study
SD: Standard deviation
SE: Standard error
SNP: Single nucleotide polymorphism

## References

[1] Lowrey PL, Takahashi JS. Genetics of circadian rhythms in Mammalian model organisms. Adv Genet. 2011;74:175–230. 21924978 10.1016/B978-0-12-387690-4.00006-4 PMC3709251

[2] Reppert SM, Weaver DR. Coordination of circadian timing in mammals. Nature. 2002;418(6901):935–41. 12198538 10.1038/nature00965

[3] Hudec M, Dankova P, Solc R, Bettazova N, Cerna M. Epigenetic regulation of circadian rhythm and its possible role in diabetes mellitus. Int J Mol Sci. 2020;21(8):3005. 10.3390/ijms21083005. 32344535 10.3390/ijms21083005 PMC7215839

[4] Schernhammer ES, Laden F, Speizer FE, Willett WC, Hunter DJ, Kawachi I, Colditz GA. Rotating night shifts and risk of breast cancer in women participating in the nurses’ health study. J Natl Cancer Inst. 2001;93(20):1563–8. 11604480 10.1093/jnci/93.20.1563

[5] Pan A, Schernhammer ES, Sun Q, Hu FB. Rotating night shift work and risk of type 2 diabetes: two prospective cohort studies in women. PLoS Med. 2011;8(12): e1001141. 22162955 10.1371/journal.pmed.1001141 PMC3232220

[6] Eurofound. European Working Condition Survey 2015 [updated 20 November 2023. Available from: https://www.eurofound.europa.eu/en/data-catalogue/european-working-conditions-survey.

[7] Eurofound. European Working Conditions Thelephone Survey 2021 [updated 20 November 2023. Available from: https://www.eurofound.europa.eu/en/data-catalogue/european-working-conditions-telephone-survey-2021-0.

[8] Mahoney MM. Shift work, jet lag, and female reproduction. Int J Endocrinol. 2010;2010: 813764. 20224815 10.1155/2010/813764 PMC2834958

[9] Cai C, Vandermeer B, Khurana R, Nerenberg K, Featherstone R, Sebastianski M, Davenport MH. The impact of occupational shift work and working hours during pregnancy on health outcomes: a systematic review and meta-analysis. Am J Obstet Gynecol. 2019;221(6):563–76. 31276631 10.1016/j.ajog.2019.06.051

[10] Knutsson A. Health disorders of shift workers. Occup Med (Lond). 2003;53(2):103–8. 12637594 10.1093/occmed/kqg048

[11] Bakulski KM, Blostein F, London SJ. Linking prenatal environmental exposures to lifetime health with epigenome-wide association studies: state-of-the-science review and future recommendations. Environ Health Perspect. 2023;131(12): 126001. 38048101 10.1289/EHP12956 PMC10695268

[12] Joubert BR, den Dekker HT, Felix JF, Bohlin J, Ligthart S, Beckett E, et al. Maternal plasma folate impacts differential DNA methylation in an epigenome-wide meta-analysis of newborns. Nat Commun. 2016;7:10577. 26861414 10.1038/ncomms10577 PMC4749955

[13] Joubert BR, Felix JF, Yousefi P, Bakulski KM, Just AC, Breton C, et al. DNA methylation in newborns and maternal smoking in pregnancy: genome-wide consortium meta-analysis. Am J Hum Genet. 2016;98(4):680–96. 27040690 10.1016/j.ajhg.2016.02.019 PMC4833289

[14] Sharp GC, Salas LA, Monnereau C, Allard C, Yousefi P, Everson TM, et al. Maternal BMI at the start of pregnancy and offspring epigenome-wide DNA methylation: findings from the pregnancy and childhood epigenetics (PACE) consortium. Hum Mol Genet. 2017;26(20):4067–85. 29016858 10.1093/hmg/ddx290 PMC5656174

[15] Varcoe TJ, Gatford KL, Kennaway DJ. Maternal circadian rhythms and the programming of adult health and disease. Am J Physiol Regul Integr Comp Physiol. 2018;314(2):R231–41. 29141950 10.1152/ajpregu.00248.2017

[16] Chaves I, van der Eerden B, Boers R, Boers J, Streng AA, Ridwan Y, et al. Gestational jet lag predisposes to later-life skeletal and cardiac disease. Chronobiol Int. 2019;36(5):657–71. 30793958 10.1080/07420528.2019.1579734

[17] Mendez N, Halabi D, Spichiger C, Salazar ER, Vergara K, Alonso-Vasquez P, et al. Gestational chronodisruption impairs circadian physiology in rat male offspring, increasing the risk of chronic disease. Endocrinology. 2016;157(12):4654–68. 27802074 10.1210/en.2016-1282

[18] Varcoe TJ, Wight N, Voultsios A, Salkeld MD, Kennaway DJ. Chronic phase shifts of the photoperiod throughout pregnancy programs glucose intolerance and insulin resistance in the rat. PLoS ONE. 2011;6(4): e18504. 21494686 10.1371/journal.pone.0018504 PMC3071829

[19] Clarkson-Townsend DA, Everson TM, Deyssenroth MA, Burt AA, Hermetz KE, Hao K, et al. Maternal circadian disruption is associated with variation in placental DNA methylation. PLoS ONE. 2019;14(4): e0215745. 31026301 10.1371/journal.pone.0215745 PMC6485638

[20] Felix JF, Joubert BR, Baccarelli AA, Sharp GC, Almqvist C, Annesi-Maesano I, et al. Cohort profile: pregnancy and childhood epigenetics (PACE) consortium. Int J Epidemiol. 2018;47(1):22–3. 29025028 10.1093/ije/dyx190 PMC5837319

[21] Boyd A, Golding J, Macleod J, Lawlor DA, Fraser A, Henderson J, et al. Cohort profile: the ‘children of the 90s’–the index offspring of the avon longitudinal study of parents and children. Int J Epidemiol. 2013;42(1):111–27. 22507743 10.1093/ije/dys064 PMC3600618

[22] Fraser A, Macdonald-Wallis C, Tilling K, Boyd A, Golding J, Davey Smith G, et al. Cohort profile: the avon longitudinal study of parents and children: ALSPAC mothers cohort. Int J Epidemiol. 2013;42(1):97–110. 22507742 10.1093/ije/dys066 PMC3600619

[23] Schisterman EF, Silver RM, Lesher LL, Faraggi D, Wactawski-Wende J, Townsend JM, et al. Preconception low-dose aspirin and pregnancy outcomes: results from the EAGeR randomised trial. Lancet. 2014;384(9937):29–36. 24702835 10.1016/S0140-6736(14)60157-4 PMC4181666

[24] Kooijman MN, Kruithof CJ, van Duijn CM, Duijts L, Franco OH, van Izendoorn MH, et al. The Generation R Study: design and cohort update 2017. Eur J Epidemiol. 2016;31(12):1243–64. 28070760 10.1007/s10654-016-0224-9 PMC5233749

[25] Guxens M, Ballester F, Espada M, Fernandez MF, Grimalt JO, Ibarluzea J, et al. Cohort profile: the INMA–INfancia y Medio Ambiente–(environment and childhood) project. Int J Epidemiol. 2012;41(4):930–40. 21471022 10.1093/ije/dyr054

[26] Magnus P, Birke C, Vejrup K, Haugan A, Alsaker E, Daltveit AK, et al. Cohort profile update: the norwegian mother and child cohort study (MoBa). Int J Epidemiol. 2016;45(2):382–8. 27063603 10.1093/ije/dyw029

[27] Magnus P, Irgens LM, Haug K, Nystad W, Skjærven R, Stoltenberg C. Cohort profile: the Norwegian mother and child cohort study (MoBa). Int J Epidemiol. 2006;35(5):1146–50. 10.1093/ije/dyl170. 16926217 10.1093/ije/dyl170

[28] Ronningen KS, Paltiel L, Meltzer HM, Nordhagen R, Lie KK, Hovengen R, et al. The biobank of the Norwegian mother and child cohort study: a resource for the next 100 years. Eur J Epidemiol. 2006;21(8):619–25. 17031521 10.1007/s10654-006-9041-x PMC1820840

[29] Send TS, Gilles M, Codd V, Wolf I, Bardtke S, Streit F, et al. Telomere length in newborns is related to maternal stress during pregnancy. Neuropsychopharmacology. 2017;42(12):2407–13. 28397798 10.1038/npp.2017.73 PMC5645750

[30] Witt SH, Frank J, Gilles M, Lang M, Treutlein J, Streit F, et al. Impact on birth weight of maternal smoking throughout pregnancy mediated by DNA methylation. BMC Genomics. 2018;19(1):290. 29695247 10.1186/s12864-018-4652-7 PMC5922319

[31] Ghosh D, Vogt A. Outliers: an evaluation of methodologies. American Statistical Association. 2012:3455- 60.

[32] Gervin K, Salas LA, Bakulski KM, van Zelm MC, Koestler DC, Wiencke JK, et al. Systematic evaluation and validation of reference and library selection methods for deconvolution of cord blood DNA methylation data. Clin Epigenetics. 2019;11(1):125. 31455416 10.1186/s13148-019-0717-y PMC6712867

[33] Leek JT, Johnson WE, Parker HS, Jaffe AE, Storey JD. The sva package for removing batch effects and other unwanted variation in high-throughput experiments. Bioinformatics. 2012;28(6):882–3. 22257669 10.1093/bioinformatics/bts034 PMC3307112

[34] Van der Most PJ, Kupers LK, Snieder H, Nolte I. QCEWAS: automated quality control of results of epigenome-wide association studies. Bioinformatics. 2017;33(8):1243–5. 28119308 10.1093/bioinformatics/btw766

[35] Willer CJ, Li Y, Abecasis GR. METAL: fast and efficient meta-analysis of genomewide association scans. Bioinformatics. 2010;26(17):2190–1. 20616382 10.1093/bioinformatics/btq340 PMC2922887

[36] isglobal-brge. EASIER: Tools for methylation data analysis. 2022 [Available from: https://github.com/isglobal-brge/EASIER.

[37] Magi R, Morris AP. GWAMA: software for genome-wide association meta-analysis. BMC Bioinformatics. 2010;11:288. 20509871 10.1186/1471-2105-11-288 PMC2893603

[38] Chen YA, Lemire M, Choufani S, Butcher DT, Grafodatskaya D, Zanke BW, et al. Discovery of cross-reactive probes and polymorphic CpGs in the Illumina Infinium HumanMethylation450 microarray. Epigenetics. 2013;8(2):203–9. 23314698 10.4161/epi.23470 PMC3592906

[39] Naeem H, Wong NC, Chatterton Z, Hong MK, Pedersen JS, Corcoran NM, et al. Reducing the risk of false discovery enabling identification of biologically significant genome-wide methylation status using the HumanMethylation450 array. BMC Genomics. 2014;15(1):51. 24447442 10.1186/1471-2164-15-51 PMC3943510

[40] Benjamini Y, Hochberg Y. Controlling the false discovery rate: a practical and powerful approach to multiple testing. J Roy Stat Soc: Ser B (Methodol). 1995;51(1):289–300.

[41] R Core Team. R: A language and environment for statistical computing. Vienna, Austria: R Foundation for Statistical Computing; 2021.

[42] Phipson B, Maksimovic J, Oshlack A. missMethyl: an R package for analyzing data from Illumina’s HumanMethylation450 platform. Bioinformatics. 2016;32(2):286–8. 26424855 10.1093/bioinformatics/btv560

[43] Breeze CE, Reynolds AP, van Dongen J, Dunham I, Lazar J, Neph S, Vierstra J, Bourque G, Teschendorff AE, Stamatoyannopoulos JA, Beck S. eFORGE v2.0: updated analysis of cell type-specific signal in epigenomic data. Bioinformatics. 2019;35(22):4767–9. 10.1093/bioinformatics/btz456. 31161210 10.1093/bioinformatics/btz456 PMC6853678

[44] Battram T, Yousefi P, Crawford G, Prince C, Sheikhali Babaei M, Sharp G, et al. The EWAS Catalog: a database of epigenome-wide association studies. Wellcome Open Res. 2022;7:41. 35592546 10.12688/wellcomeopenres.17598.1 PMC9096146

[45] Kupers LK, Monnereau C, Sharp GC, Yousefi P, Salas LA, Ghantous A, et al. Meta-analysis of epigenome-wide association studies in neonates reveals widespread differential DNA methylation associated with birthweight. Nat Commun. 2019;10(1):1893. 31015461 10.1038/s41467-019-09671-3 PMC6478731

[46] Merid SK, Novoloaca A, Sharp GC, Kupers LK, Kho AT, Roy R, et al. Epigenome-wide meta-analysis of blood DNA methylation in newborns and children identifies numerous loci related to gestational age. Genome Med. 2020;12(1):25. 32114984 10.1186/s13073-020-0716-9 PMC7050134

[47] Vehmeijer FOL, Kupers LK, Sharp GC, Salas LA, Lent S, Jima DD, et al. DNA methylation and body mass index from birth to adolescence: meta-analyses of epigenome-wide association studies. Genome Med. 2020;12(1):105. 33239103 10.1186/s13073-020-00810-w PMC7687793

[48] Wackers P, Dolle MET, van Oostrom CTM, van Kerkhof LWM. Exploration of genome-wide DNA methylation profiles in night shift workers. Epigenetics. 2023;18(1):2152637. 36457290 10.1080/15592294.2022.2152637 PMC9980630

[49] Sammallahti S, Koopman-Verhoeff ME, Binter AC, Mulder RH, Cabre-Riera A, Kvist T, et al. Longitudinal associations of DNA methylation and sleep in children: a meta-analysis. Clin Epigenetics. 2022;14(1):83. 35790973 10.1186/s13148-022-01298-4 PMC9258202

[50] Gaunt TR, Shihab HA, Hemani G, Min JL, Woodward G, Lyttleton O, et al. Systematic identification of genetic influences on methylation across the human life course. Genome Biol. 2016;17:61. 27036880 10.1186/s13059-016-0926-z PMC4818469

[51] Min JL, Hemani G, Hannon E, Dekkers KF, Castillo-Fernandez J, Luijk R, et al. Genomic and phenotypic insights from an atlas of genetic effects on DNA methylation. Nat Genet. 2021;53(9):1311–21. 34493871 10.1038/s41588-021-00923-x PMC7612069

[52] Maechler M. diptest 2024 [Available from: https://github.com/mmaechler/diptest?tab=readme-ov-file#readme.

[53] Ruiz-Arenas C, Hernandez-Ferrer C, Vives-Usano M, Marí S, Quintela I, Mason D, et al. Identification of autosomal cis expression quantitative trait methylation (cis eQTMs) in children’s blood. Elife. 2022. 10.7554/eLife.65310. 35302492 10.7554/eLife.65310 PMC8933004

[54] Boers R, Boers J, de Hoon B, Kockx C, Ozgur Z, Molijn A, et al. Genome-wide DNA methylation profiling using the methylation-dependent restriction enzyme LpnPI. Genome Res. 2018;28(1):88–99. 29222086 10.1101/gr.222885.117 PMC5749185

[55] El Sharkawy M, Felix JF, Grote V, Voortman T, Jaddoe VWV, Koletzko B, Kupers LK. Animal and plant protein intake during infancy and childhood DNA methylation: a meta-analysis in the NutriPROGRAM consortium. Epigenetics. 2024;19(1):2299045. 38198623 10.1080/15592294.2023.2299045 PMC10793674

[56] Fragoso-Bargas N, Page CM, Joubert BR, London SJ, Lee-Odegard S, Opsahl JO, et al. Epigenome-wide association study of serum folate in maternal peripheral blood leukocytes. Epigenomics. 2023;15(1):39–52. 36974632 10.2217/epi-2022-0427 PMC10072132

[57] Lee MK, Xu CJ, Carnes MU, Nichols CE, Ward JM, Consortium B, et al. Genome-wide DNA methylation and long-term ambient air pollution exposure in Korean adults. Clin Epigenetics. 2019;11(1):37. 30819252 10.1186/s13148-019-0635-z PMC6396524

[58] Mulder RH, Neumann A, Cecil CAM, Walton E, Houtepen LC, Simpkin AJ, et al. Epigenome-wide change and variation in DNA methylation in childhood: trajectories from birth to late adolescence. Hum Mol Genet. 2021;30(1):119–34. 33450751 10.1093/hmg/ddaa280 PMC8033147

[59] Islam SA, Goodman SJ, MacIsaac JL, Obradovic J, Barr RG, Boyce WT, Kobor MS. Integration of DNA methylation patterns and genetic variation in human pediatric tissues help inform EWAS design and interpretation. Epigenetics Chromatin. 2019;12(1):1. 30602389 10.1186/s13072-018-0245-6 PMC6314079

[60] Li Y, Chen JA, Sears RL, Gao F, Klein ED, Karydas A, Geschwind MD, Rosen HJ, Boxer AL, Guo W, Pellegrini M, Horvath S, Miller BL, Geschwind DH, Coppola G. An epigenetic signature in peripheral blood associated with the haplotype on 17q21.31, a risk factor for neurodegenerative tauopathy. PLoS Genet. 2014;10(3):e1004211. 10.1371/journal.pgen.1004211. 24603599 10.1371/journal.pgen.1004211 PMC3945475

[61] Spiers H, Hannon E, Schalkwyk LC, Smith R, Wong CC, O’Donovan MC, et al. Methylomic trajectories across human fetal brain development. Genome Res. 2015;25(3):338–52. 25650246 10.1101/gr.180273.114 PMC4352878

[62] Aghagoli G, Sheinkopf SJ, Everson TM, Marsit CJ, Lee H, Burt AA, et al. Epigenome-wide analysis identifies genes and pathways linked to acoustic cry variation in preterm infants. Pediatr Res. 2021;89(7):1848–54. 32967004 10.1038/s41390-020-01172-0 PMC7985041

[63] Bhatti P, Zhang Y, Song X, Makar KW, Sather CL, Kelsey KT, et al. Nightshift work and genome-wide DNA methylation. Chronobiol Int. 2015;32(1):103–12. 25187986 10.3109/07420528.2014.956362

[64] Breton CV, Marsit CJ, Faustman E, Nadeau K, Goodrich JM, Dolinoy DC, et al. Small-magnitude effect sizes in epigenetic end points are important in children’s environmental health studies: the children’s environmental health and disease prevention research center’s epigenetics working group. Environ Health Perspect. 2017;125(4):511–26. 28362264 10.1289/EHP595 PMC5382002

[65] Horani A, Druley TE, Zariwala MA, Patel AC, Levinson BT, Van Arendonk LG, et al. Whole-exome capture and sequencing identifies HEATR2 mutation as a cause of primary ciliary dyskinesia. Am J Hum Genet. 2012;91(4):685–93. 23040496 10.1016/j.ajhg.2012.08.022 PMC3484505

[66] Bogershausen N, Shahrzad N, Chong JX, von Kleist-Retzow JC, Stanga D, Li Y, et al. Recessive TRAPPC11 mutations cause a disease spectrum of limb girdle muscular dystrophy and myopathy with movement disorder and intellectual disability. Am J Hum Genet. 2013;93(1):181–90. 23830518 10.1016/j.ajhg.2013.05.028 PMC3710757

[67] Koehler K, Milev MP, Prematilake K, Reschke F, Kutzner S, Juhlen R, et al. A novel TRAPPC11 mutation in two Turkish families associated with cerebral atrophy, global retardation, scoliosis, achalasia and alacrima. J Med Genet. 2017;54(3):176–85. 27707803 10.1136/jmedgenet-2016-104108

[68] Cushing KC, Chen Y, Du X, Chen V, Kuppa A, Higgins P, Speliotes EK. Risk variants in or near ZBTB40 AND NFATC1 increase the risk of both IBD and adverse bone health outcomes highlighting common genetic underpinnings across both diseases. Inflamm Bowel Dis. 2023;29(6):938–45. 36680554 10.1093/ibd/izac273 PMC10465078

[69] Biason-Lauber A, Konrad D, Navratil F, Schoenle EJ. A WNT4 mutation associated with Mullerian-duct regression and virilization in a 46. XX woman N Engl J Med. 2004;351(8):792–8. 15317892 10.1056/NEJMoa040533

[70] Mandel H, Shemer R, Borochowitz ZU, Okopnik M, Knopf C, Indelman M, et al. SERKAL syndrome: an autosomal-recessive disorder caused by a loss-of-function mutation in WNT4. Am J Hum Genet. 2008;82(1):39–47. 18179883 10.1016/j.ajhg.2007.08.005 PMC2253972

[71] Barker DJ, Osmond C, Forsen TJ, Kajantie E, Eriksson JG. Trajectories of growth among children who have coronary events as adults. N Engl J Med. 2005;353(17):1802–9. 16251536 10.1056/NEJMoa044160

[72] Everson TM, Vives-Usano M, Seyve E, Cardenas A, Lacasana M, Craig JM, et al. Placental DNA methylation signatures of maternal smoking during pregnancy and potential impacts on fetal growth. Nat Commun. 2021;12(1):5095. 34429407 10.1038/s41467-021-24558-y PMC8384884

[73] Fernandez-Jimenez N, Fore R, Cilleros-Portet A, Lepeule J, Perron P, Kvist T, et al. A meta-analysis of pre-pregnancy maternal body mass index and placental DNA methylation identifies 27 CpG sites with implications for mother-child health. Commun Biol. 2022;5(1):1313. 36446949 10.1038/s42003-022-04267-y PMC9709064

[74] Olsson Lindvall M, Angerfors A, Andersson B, Nilsson S, Davila Lopez M, Hansson L, et al. Comparison of DNA methylation profiles of hemostatic genes between liver tissue and peripheral blood within individuals. Thromb Haemost. 2021;121(5):573–83. 33202445 10.1055/s-0040-1720980 PMC8116175

