# Supplementary material for: Associations of maternal night shift work during pregnancy with DNA methylation in offspring: a meta-analysis in the PACE consortium
Source: Clin Epigenetics. 2025 Jan 22;17:12. doi: 10.1186/s13148-024-01810-y (PMC11756212; doi:10.1186/s13148-024-01810-y)
Supplement: Supplementary file 1 — Additional file1 (DOCX 98 KB) [file 13148_2024_1810_MOESM1_ESM.docx]

# Cohort-specific Methods

# THE AVON LONGITUDINAL STUDY OF PARENTS AND CHILDREN (ALSPAC)

# Study description

ALSPAC is a prospective cohort study based in the South West of England, UK (1, 2). Pregnant women resident in Avon, UK, with expected dates of delivery 1st April 1991 to 31st December 1992 were invited to take part in the study. The initial number of pregnancies enrolled is 14,541 (for these at least one questionnaire has been returned or a “Children in Focus” clinic had been attended by 19/07/99). When the oldest children were approximately 7 years of age, an attempt was made to bolster the initial sample with eligible cases who had failed to join the study originally. The total sample size after the age of seven is 15,447 pregnancies, resulting in 15,658 foetuses. Detailed information has been collected on these women, their partners and their offspring at regular intervals to the present date. Ethical approval for the study was obtained from the ALSPAC Ethics and Law Committee and the Local Research Ethics Committees. Informed consent for the use of data collected via questionnaires and clinics was obtained from participants following the recommendations of the ALSPAC Ethics and Law Committee at the time. Please note that the study website contains details of all the data that is available through a fully searchable data dictionary and variable search tool: http://www.bristol.ac.uk/alspac/researchers/our-data/.

# Methods

Exposure

Information on mothers’ night shift work during pregnancy was obtained in questionnaires administered at 18 and 32 weeks of gestation. Night shift work was assessed based on the questions “Do you do shift work? If yes, does this include night shifts” asked to mothers at 32 weeks of gestation, and the question “Do/did you do shift work? For those who reported that they had worked during pregnancy. If yes, does this include night shifts?” asked to mothers at 18 weeks of gestation. Mothers who indicated night shifts in at least one of the questions were classed as night shift workers (81 (9.9%) out of 818 mothers).

DNA methylation data

DNA extraction, wet laboratory preparation and DNA methylation measurement were performed as part of the ARIES project, as described previously (3, 4) Briefly, samples from all ARIES time-points were distributed semi-randomly across HM450 slides to minimise the potential for confounding by technical batch. Data pre-processing was performed using the meffil package (3). Samples failing quality control (average probe detection p value ≥ 0.01, those with sex or genotype mismatches) were excluded from further analysis, and probes containing < 95% of signals detectable above background signal (detection p value < 0.01) were also removed. Functional normalization was performed to minimize non-biological variation in probes.

Covariates

Child sex, gestational age, and birth weight were recorded in the delivery room and abstracted from obstetric records and/or birth notifications. Gestational age at birth was calculated based on the date of the mother’s last menstrual period or from ultrasound. Maternal educational attainment reported during pregnancy was categorized into low (education up to the age of 16 years), medium (education up to the age of 18 years) and high (university education). Maternal age at delivery was derived from the mother’s date of birth, which was recorded at the time of recruitment, and the date of birth of her offspring. Information on mother’s smoking status during pregnancy was obtained in a questionnaire administered at 18 weeks of gestation. Maternal self-reported smoking during pregnancy was categorized into no smoking, smoking in the first trimester only and smoking which continued after the first trimester. After enrolment, the mother was asked to record her height and pre-pregnancy weight in a questionnaire administered at 12 weeks gestation, from which pre-pregnancy body mass index (BMI) was calculated. The Bakulski cord blood reference panel (5) was used to estimate cell composition. Adjustment for batch effects was done by surrogate variables for technical batch, using the sva package in R (6). Given the low ethnic diversity in the sample (<3% self-identified as non-white), we did not include ethnicity as a covariate.

# EAGeR

# Study description

EAGeR was a multicenter, double-blind clinical trial that randomized women to low-dose aspirin or placebo prior conception (2007-2011; NCT00467363) (7). Women between 18 and 40 years old, with a history of one or two prior pregnancy losses, no history of infertility, actively trying to conceive and with regular menstrual cycles of 21-42 days during the past year, were eligible for the trial. Women were followed for up to six menstrual cycles while attempting pregnancy and then throughout pregnancy for women who conceived. Among women who conceived during follow-up, the trial collected 10 ml of cord blood from over 90% of deliveries at the Utah study site. The study was approved by the institutional review board (IRB) at the University of Utah (Salt Lake City, Utah IRB #1002521), and all participants provided written informed consent prior to enrolling.

# Methods

Exposure

Night work was defined as "work in which most of the hours (>50%) are in the evening (between 4pm and midnight) or at night (between midnight and 8 a.m.)". On the preconception baseline questionnaire (approximately 2 months prior to pregnancy) women were asked: "Have your jobs involved exposure to any of the following?". Women were instructed to select Yes/No for "Night work". Women who selected “yes” were classified as exposed to shift work.

DNA methylation data

DNA was extracted, and DNA methylation was profiled using the EPIC BeadChip (n=391). Among the 391 newborns with DNA methylation data, we excluded 33 newborns who were not of non-Hispanic white race/ethnicity to avoid population stratification in the meta-analysis. Of note, randomization to low-dose aspirin had no impact on DNA methylation in cord blood in this study population (8). DNA methylation data were processed using the minfi package (9) in R to identify failed probes and perform scaling with Illumina control probes to determine methylation values. The beta value was determined for each of the CpG sites by the fluorescent signals. Background and dye-bias corrections were applied. Beta values were normalized using quantile normalization to address potential probe type bias. Principal component analysis (PCA) was performed to identify and exclude samples mismatched on sex. We extracted the detection p value for each methylation measure (per site per sample). We removed samples with low passing rate (< 97%) based on detection p value (p > 0.01) and bead counts (<3).

Covariates

Maternal age in years (continuous), maternal education (=< High School or > High School), maternal smoking status (never or ever), and maternal pre-pregnancy BMI in kg/m^2^ (continuous) were captured on the baseline questionnaire prior to pregnancy. Additional covariates were abstracted from medical records and included child sex (male or female), offspring gestational age in weeks (continuous) and offspring birth weight in grams (continuous). Estimated cell types (B cell, Monocytes, CD4T, CD8T, granulocytes, natural killer and nucleated red blood cells) were estimated using the Salas reference set (10). To account for batch effects we adjusted for plate in the models. Participant ethnicity was determined by self-report.

**Generation R**

# Study description

Generation R Study is a population-based prospective birth-cohort from fetal life onwards established in Rotterdam, the Netherlands (11). The Medical Ethical Committee of Erasmus MC, University Medical Center Rotterdam, approved the study (MEC 198.782/2001 /31). Women with an expected delivery date between April 2002 and January 2006 living in Rotterdam were eligible to enroll the study, and written informed consent was obtained from all participants. In 1396 of the 9901 live-born newborns participating in the Generation R Study, we measured genome-wide DNA methylation in cord blood. This subgroup was selected as a relatively homogeneous, European-ancestry subgroup based on genetic information. A total of 13 mothers had two (non-twin) children. Per mother, we included only one child. Three children were excluded due to missing information for maternal smoking status, and 10 were randomly excluded. We performed complete-case analysis, and a total of 1274 mother–newborn pairs had complete information on the exposure and DNA methylation.

# Methods

Exposure

Maternal working conditions were assessed using a questionnaire, sent to the mother in late pregnancy (>25week) (11). Shift work was assessed with a multiple choice question: “Which of the following activities have you done in the past 3 months? Night duty” The possible answers were: (a) seldom or never; (b) occasionally; (c) fairly often; (d) very frequently. Mothers were classified as exposed if they selected b, c, or d.

DNA methylation data

We used the salting-out method to extract DNA from cord blood samples. Five-hundred nanograms of DNA were bisulfite converted using the EZ-96 DNA Methylation kit (Shallow) (Zymo Research Corporation, Irvine, USA). Samples were processed with the Illumina Infinium HumanMethylation450 BeadChip (Illumina Inc., San Diego, USA). Quality control and normalization were performed using the CPACOR workflow (12). Probes with a detection p ≥ 1E^−16^ were set to missing. Intensity values were quantile normalized. We removed arrays with technical problems, a call rate ≤ 95%, or a mismatch between the expected sex of participant and sex determined by chromosome X and Y probe intensities. Probes on the sex chromosomes were removed before the analyses. We used untransformed beta values as measures of DNA methylation, and the final DNA methylation dataset contained information on 458,563 CpGs.

Covariates

Child sex and birth weight were obtained from midwife and hospital registries. Clinical gestational age was determined at the first visit to the research center during pregnancy (13). Clinical gestational age was based on a known and reliable first day of the last menstrual period, and a regular menstrual cycle of 28 ± 4 days. If mothers did not know the exact date of their last menstrual period, or had an irregular menstrual cycle, we established gestational age by ultrasound examination. Maternal education was self-reported and defined based on the highest level of education finished by the mothers. This covariate was dichotomized into low and medium education versus higher education. Maternal age was reported by the mother at intake. Maternal smoking during pregnancy was self-reported through repeated questionnaires in pregnancy as “no smoking during pregnancy”, “smoked but quit before second trimester”, or “smoked throughout pregnancy”. We recoded these categories into no smoking and quitting when pregnancy was known versus sustained smoking. Maternal BMI was calculated based on self-reported height and weight measured at our research center in early pregnancy. Plate number was included as batch variable. Cell type proportions were estimated using a cord blood specific reference that estimates the proportions of white blood cell subtypes CD8+ T cells, CD4+ T cells, natural killer cells, B cells, monocytes, granulocytes, and nucleated red blood cells in cord blood (10). Analyses were restricted to European ancestry determined based on genetic information.

**INfancia y Medio Ambiente (INMA)**

# Study description

The INfancia y Medio Ambiente (INMA, Environment and Childhood) Project is a network of Spanish birth cohorts that aim to study the role of environmental pollutants in air, water and diet during pregnancy and early childhood in relation to child growth and development (14). All participating parents provided written informed consents. The study was approved by the Ethical Committee of the Municipal Institute of Medical Investigation and by the Ethical Committee of the hospitals involved in the study.

# Methods

Exposure

Women self-reported occupational exposures at 32 weeks of gestation. They were asked about their work schedule from one month before getting pregnant to 32 weeks of gestation. The possible answers were: fixed schedule (N=330), fixed schedule at night (N=6), shift work without night work (N=1), shift work with night work (N=7), or other (N=2, "17h a 1h de la madrugada" and "horario 24h en el domicilio de la anciana"). We categorized maternal into shift work if they selected “fixed schedule at night”, “shift work with night work” and “other”. “Fixed schedule” and “shift work without night work” were set to the control group.

DNA methylation data

Cord blood DNA was extracted using the Chemagen kit (Perkin Elmer). DNA concentration was determined by a NanoDrop spectrophotometer (Thermo Scientific) and with the Quant-iT PicoGreen dsDNA Assay Kit (Life Technologies). DNA methylation data was produced in two laboratories: the Genome Analysis Facility of the University Medical Center Groningen (UMCG) in Holland as part of the MeDALL project, and the Bellvitge Biomedical Research Institute (IDIBELL) in Barcelona as part of the BREATHE project. Both laboratories randomized the samples in batches and followed the Illumina protocol for the Infinium HumanMethylation450 BeadChip. Briefly, 500 ng of DNA was bisulfite-converted using the EZ 96-DNA methylation kit, and DNA methylation was measured through hybridization on the BeadChips. BeadChips were scanned with an Illumina iScan and image data was uploaded into the Methylation Module of Illumina’s analysis software GenomeStudio, and converted in beta-values. We preprocessed DNA methylation data using the minfi package (9). First, we removed 2 samples with bad overall quality or with low detection p-value according to the output of the MethylAid package (15). Then, we removed 3 samples whose sex was wrongly predicted using shinyMethyl (16). Following guidelines of Lehne work (12), we increased the stringency of the detection p-value threshold to 10-^16^ and we filtered 18 samples with a call rate <98%. We normalized data with functional normalization. Correlation between SNP in replicates samples was checked and probes not measuring SNPs were discarded. Finally, duplicated samples were removed, prioritizing MeDALL samples over BREATHE samples.

Covariates

Child sex and birth weight were obtained from medical records. We calculated gestational age from the date of the last menstrual period (LMP) reported at recruitment and confirmed using estimates based on ultrasound examination in the 12th week of gestation. When the difference between the LMP reported at recruitment and estimated from the ultrasound was ≥ 7 days, then LMP was estimated using a quadratic regression formula defined by (17). Maternal age was recorded at inclusion, by questionnaire. At inclusion (10-13 WG), women reported their height and weight before pregnancy, and we calculated maternal BMI from these records. Maternal education was assessed at child birth. We categorized maternal education into high (University level or higher), medium (secondary education), or low (primary education, lower or no education). We classified maternal self-reported smoking status during pregnancy into no smoking or smoking stopped in early-pregnancy versus smoking throughout pregnancy. We estimated blood cell type proportions from methylation data, applying the Houseman method (18) reference panel. We applied ComBat to remove batch effect (19). European ancestry was defined based on self-reported information.

# Norwegian Mother, Father and Child Cohort Study (MoBa1 and MoBa2)

# Study description

For MoBa1, mother-offspring pairs were selected from a sub-study within the Norwegian Mother, Father and Child Cohort Study (MoBa) (20-22). The sub-study was a sample of asthma cases at age three years and a cohort random sample without asthma at age three years (23). Offspring in this study were born in 2002 to 2004. A non-overlapping subset was selected from MoBa (20-22), refered to as MoBa2, which included a sample of asthma cases at age seven years and non-asthmatic controls (24). Offspring in this subset were born in 2000 to 2005. MoBa1 and MoBa2 were approved by the Regional Committee for Ethics in Medical Research in Norway and the Institutional Review Board of the National Institute of Environmental Health Sciences in the USA. These analyses were analyzed using data release version 12. This study included European infants with maternal data on working hours during pregnancy, blood DNA methylation measurements at birth, and complete covariate data (n=946 in MoBa1 and n=583 in MoBa2). Each dataset was analyzed separately.

# Methods

Exposure

Around the 18^th^ week of gestation, women reported their working hours: permanent day work, permanent afternoon or evening work, permanent night work, shift work or shift rotations, no set times, or other. Women were able to select more than one option. Women were defined as exposed to shift work if they selected permanent night work or shift work or shift rotations and did not select permanent day work or permanent afternoon or evening work. Women who selected at least one option were considered exposed to shift work (68 missing in MoBa1, 68 missing in MoBa2).

DNA methylation data

Methylation was measured in MoBa1 first (25). The same laboratory measured DNA methylation in MoBa2 later (26). Details of how DNA methylation were measured and the quality control procedure for MoBa1 and MoBa2 have been previously described (26). The same procedures were applied to both studies. Briefly, samples of umbilical cord blood were collected at birth and stored at -80°C. DNA was bisulfite converted using the EZ-96 DNA Methylation kit (Zymo Research Corporation, Irvine, CA, USA). DNA methylation was assessed at 485,577 CpGs using Illumina’s Infinium HumanMethylation450 BeadChip (27). We used the minfi package (9) in R to calculate the beta values at each CpG. Quality control procedures were applied on the beta methylation values. We excluded 65 control probes, probes on the X chromosome (N=11,230) and probes on the Y chromosome (N=416). CpGs missing >10% of methylation values were removed (20 CpGs in MoBa1, 0 CpGs in MoBa2). We excluded samples identified by Illumina to have failed or those with an average detection p-value <0.05 across all probes (49 in MoBa1, 35 in MoBa2), as well as samples with sex mismatches (13 in MoBa1, 8 in MoBa2). We used the beta mixture quantile (BMIQ) to normalize the data (28). Extreme beta methylation values were winsorized to the value at the upper and lower 1%. A total of 1,068 samples passed quality control in MoBa1 and 685 samples passed quality control in MoBa2. Genotype data were measured using Illumina HumanCore. We ran principal components to identify and exclude samples that were ancestry outliers (12 in MoBa1, 5 in MoBa2). The final DNA methylation dataset contained information on 473,844 (MoBa1) and 473,748 (MoBa2) probes.

Covariates

MoBa participants completed the first questionnaire around the 17^th^ week of gestation and self-reported their age, education, pre-pregnancy weight, and height. MoBa did not collect information on **maternal age** at conception, so we used maternal age at birth. Maternal education was defined as: 1) less than high school, 2) completed high school, 3) some college, or 4) college or higher. Maternal pre-pregnancy BMI was calculated from self-reported weight and height, and extreme values were removed. Maternal smoking status was determined based on measured cotinine levels around the 18^th^ week of gestation and self-reported smoking information was collected at the 18^th^ week of gestation, 30rd week of gestation, and 6th month after birth. Participants were categorized as either 1) no smoking or quit during pregnancy or 2) sustained smoking. MoBa participants were linked to the Medical Birth Registry of Norway to collect information on maternal age, infant sex, birth weight, and gestational age at birth. We used ComBat from the sva package in R for batch correction (19). All included children were from European ancestry. All models were also adjusted for the selection factor. In MoBa1, the selection factor was asthma status at 3 years old (yes/no). In MoBa2, participants were selected into the study because of one of the following three groups: asthma status at 7 years old (yes/no) or had folate measured regardless of asthma status. Cell type proportions were estimated using the Houseman method (18) with the Gervin et al reference panel with IDOL optimization (10). Restriction to European ancestry was based on genetic information.

**POSEIDON**

# Study description

POSEIDON (Pre-, Peri-, and Postnatal Stress: Epigenetic Impact on DepressiON) is a study on stress and health, which collected mother-child dyads in obstetric hospitals in the Rhine-Neckar Region of Germany, initially from 2010 to 2017. A reassessment with an online questionnaire was done in 2020. Mothers were included in the study 4-8 weeks prior to delivery (T1), all participants gave their written informed consent. Ethics approval was given by the by Ethics Committee of the Medical Faculty Mannheim of the University of Heidelberg. The second assessment (T2) took place at childbirth followed by two assessments 6 (T3) and 45 months (T4) after birth. Mothers and children were included in the study based on several factors described elsewhere (29, 30).

# Methods

Exposure

At T1, mothers were asked if they worked shifts (“Did you work shifts or at night before your pregnancy / in early pregnancy?”) with following answer options: (1) No; (2) Yes, worked shifts, but without night shifts; (3) Yes, worked shifts, with night shifts; (4) Yes, worked only night shifts (no rotating shifts); (5) Unemployed; (6) Parental leave; (7) Student. Mothers who answered "Yes, worked shifts, with night shifts" (3) were assigned to the exposed group (1). Mothers who gave any other answer were assigned to the non-exposed group (0). There was one mother with missing information for the item.

DNA methylation data

DNA methylation levels were measured in cord blood for 313 newborns collected at childbirth with the Illumina Infinium HumanMethylation450K Beadchip. Intensity data were extracted from raw data (idat) files using an updated version of the pipeline published by (12). Intensity data were quantile normalized within subsets of probe types prior to converting to beta values. Samples with insufficient DNA quality, insufficient bisulfite conversion; or failure in detection (detection P-value > 0.01 at more than 1% of positions), sex-mismatch between phenotype and methylation data, and >4.5 standard deviations away from the mean on one of the 20 principal components for population stratification (ancestry) were excluded. Probes were excluded in case of detection p-value threshold (positions/sites) > 0.01, a call rate < 95% (samples), and X- or Y linked status. The first 10 principal components of the control probes were used to accounting for batch effects. Cell counts were estimated using a cord blood reference panel (10).

Covariates

Maternal age in years was assessed at T1. Maternal education was assessed at T1, and coded following ISCED-1997 levels and assigned to following categories: ISCED-1997 levels 0-2 assigned "lower" (1), levels 3-4 assigned "middle" (2), and levels 5-6 assigned "higher" (3) education level. Maternal smoking was assessed at T1. Mothers who smoked daily or occasionally in the third trimester were assigned "yes" (1). Individuals who never smoked or no longer smoked during the third trimester were assigned "no" (0). Maternal BMI was assessed at T1 in kg/m^2^. Child’s sex was assessed at T2 and coded 0 for male, and 1 for female. Gestational age was assessed at T2 and coded in full weeks. Birth weight was assessed at T2 and recorded in grams. To address the ancestry requirements, for the present analysis, outliers on the first 20 ancestry principal components derived from genome-wide genotype data (Illumina Psych Array) were excluded from the analysis.

# THE AVON LONGITUDINAL STUDY OF PARENTS AND CHILDREN (ALSPAC)

# Funding

The UK Medical Research Council and Wellcome (grant ref: 217065/Z/19/Z) and the University of Bristol provide core support for ALSPAC. This publication is the work of the authors and RCR will serve as guarantors for the contents of this paper. Methylation data in the ALSPAC cohort were generated as part of the UK BBSRC funded (grant numbers: BB/I025751/1 and BB/I025263/1) Accessible Resource for Integrated Epigenomic Studies (ARIES, http://www.ariesepigenomics.org.uk). D.C., work in a Unit that is supported by the University of Bristol and the UK Medical Research Council (grant number: MC_UU_00011/5). The funders had no role in the planning or execution of the study nor the interpretation or publication of its results.Data availability

The datasets supporting the results of this study are available from ALSPAC. The ALSPAC policy on data sharing is available at www.bristol.ac.uk/alspac. To discuss access to ALSPAC data, please contact the ALSPAC executive team on alspac-exec@bristol.ac.uk. Details of all available data and samples can be found using the following link (<http://www.bristol.ac.uk/alspac/researchers/our-data>).

A comprehensive list of grants funding is available on the ALSPAC website (http://www.bristol.ac.uk/alspac/external/documents/grant-acknowledgements.pdf).

# Acknowledgments

We are extremely grateful to all the families who took part in this study, the midwives for their help in recruiting them, and the whole ALSPAC team, which includes interviewers, computer and laboratory technicians, clerical workers, research scientists, volunteers, managers, receptionists and nurses.

# EAGeR

# Funding

This work was supported by the Intramural Research Program of the *Eunice Kennedy Shriver* National Institute of Child Health and Human Development (National Institutes of Health, Bethesda, MD, USA) under contract numbers HHSN267200603423, HHSN267200603424, HHSN267200603426, and HHSN275201300023I-HHSN2750008.

# Data availability

The data for the current analysis is available from the co-author (edwina.yeung@nih.gov) on reasonable request pending application and approval.

# Acknowledgments

This work utilized the computational resources of the NIH High Performance Computing Biowulf cluster (<http://hpc.nih.gov>).

# Generation R

# Funding

The general design of the Generation R Study is made possible by financial support from Erasmus MC, University Medical Centre Rotterdam, Erasmus University Rotterdam, the Netherlands Organization for Health Research and Development (ZonMw), the Netherlands Organization for Scientific Research (NWO), the Ministry of Health, Welfare and Sport, and the Ministry of Youth and Families. The EWAS data was funded by a grant to VWVJ from the Netherlands Genomics Initiative (NGI)/Netherlands Organization for Scientific Research (NWO) Netherlands Consortium for Healthy Aging (NCHA; project number 050-060-810), by funds from the Genetic Laboratory of the Department of Internal Medicine, Erasmus MC, University Medical Centre Rotterdam (R01HD068437). The project was supported by funding from the European Union’s Horizon 2020 research and innovation program under grant agreements No 733206 (LifeCycle), 874739 (LongITools) and 874583 (ATHLETE), and from the European Joint Programming Initiative ‘A Healthy Diet for a Healthy Life’ (JPI HDHL, NutriPROGRAM project, ZonMw the Netherlands no. 529051022).

# Data availability

Data from this study are available upon reasonable request to the director of the Generation R Study (generationr@erasmusmc.nl), subject to local, national and European rules and regulations.

# Acknowledgments

The Generation R Study is conducted by Erasmus MC in close collaboration with the School of Law and Faculty of Social Sciences of the Erasmus University Rotterdam, the Municipal Health Service Rotterdam area, Rotterdam, the Rotterdam Homecare Foundation, Rotterdam, and the Stichting Trombosedienst & Artsenlaboratorium Rijnmond (STAR-MDC), Rotterdam. We gratefully acknowledge the contribution of children and parents, general practitioners, hospitals, midwives, and pharmacies in Rotterdam. The generation and management of the Illumina 450K methylation array data (EWAS data) for the Generation R Study was executed by the Human Genotyping Facility of the Genetic Laboratory of the Department of Internal Medicine, Erasmus MC, and the Netherlands. We thank Mr Michael Verbiest, Ms Mila Jhamai, Ms Sarah Higgins, Mr Marijn Verkerk, and Dr Lisette Stolk for their help in creating the EWAS database. We thank Dr Alexander Teumer for his work on the quality control and normalization scripts.

**INfancia y Medio Ambiente (INMA)**

# Funding

This study was funded by grants from Instituto de Salud Carlos III (Red INMA G03/176; CB06/02/0041; FIS-FEDER-PI03-1615PI041436; FIS-FEDER-PI06/0867, PI081151 incl. FEDER funds; FIS-PI11/00610,PI12/01890 incl. FEDER funds; CP13/00054 incl. FEDER funds; PI15/00118 incl. FEDER funds; CP16/00128 incl. FEDER funds; PI16/00118 incl. FEDER funds; PI16/00261 incl. FEDER funds; PI18/00547 incl. FEDER funds, CPII18/00018), CIBERESP, Generalitat de Catalunya-CIRIT 1999SGR 00241, Generalitat de Catalunya-AGAUR (2009 SGR 501, 2014 SGR 822), Fundació La marató de TV3 (090430), Spanish Ministry of Economy and Competitiveness (SAF2012-32991 incl. FEDER funds), Agence Nationale de Securite Sanitaire de l’Alimentation de l’Environnement et du Travail (1262C0010; EST-2016 RF-21), EU Commission (261357, 308333, 603794, 634453, and 824989), and European Research Council (268479). ISGlobal acknowledges support from the Spanish Ministry of Science and Innovation through the “Centro de Excelencia Severo Ochoa 2019-2023” Program (CEX2018-000806-S), and support from the Generalitat de Catalunya through the CERCA Program.

# Data availability

The individual level data used are not publicly available. Nonetheless, individual level data can still be shared with external researchers after signature of a data transfer agreement (DTA).

# Acknowledgments

INMA researchers thank all the participants for their generous collaboration. A full roster of the INMA Project Investigators can be found at http://www.proyectoinma.org/presentacioninma/listado-investigadores/en_listado-investigadores.html.

# Norwegian Mother, Father and Child Cohort Study (MoBa1 and MoBa2)

# Funding

The Norwegian Mother and Child Cohort Study are supported by the Norwegian Ministry of Health and Care Services and the Ministry of Education and Research, NIH/NIEHS (contract no N01-ES-75558), NIH/NINDS (grant no.1 UO1 NS 047537-01 and grant no.2 UO1 NS 047537-06A1). For this work, MoBa 1 and 2 were supported by the Intramural Research Program of the NIH, National Institute of Environmental Health Sciences (Z01-ES-49019) and the Norwegian Research Council/BIOBANK (grant no 221097). This work was partly supported by the Research Council of Norway through its Centres of Excellence funding scheme, project number 262700.

# Data availability

Data from the Norwegian Mother, Father and Child Cohort Study and the Medical Birth Registry of Norway used in this study are managed by the national health register holders in Norway (Norwegian Institute of public health) and can be made available to researchers, provided approval from the Regional Committees for Medical and Health Research Ethics (REC), compliance with the EU General Data Protection Regulation (GDPR) and approval from the data owners. The consent given by the participants does not open for storage of data on an individual level in repositories or journals. Researchers who want access to data sets for replication should apply through helsedata.no. Access to data sets requires approval from The Regional Committee for Medical and Health Research Ethics in Norway and an agreement with MoBa. Access to the MoBa1 and MoBa2 data is available upon application to the Norwegian Institute of Public Health (NIPH). An application form in English can be found at the NIPH website (<http://www.fhi.no/en/>).

# Acknowledgments

We are grateful to all the participating families in Norway who take part in this on-going cohort study. We thank Jakob Mjånes and Elin Alsaker of the National Institute of Public Health (Bergen, Norway), Dr. Frank Day of NIEHS and Dr. Jianping Jin of Westat (Durham, NC) for expert data management and computational assistance.

**POSEIDON**

# Funding

This study was supported by a grant of the Dietmar Hopp Foundation

# Data availability

Access to data can be requested on reasonable demand. Data sharing is subject to national and European rules and regulations (Stephanie Witt: stephanie.witt@zi-mannheim.de).

# Acknowledgments

We thank all parents and children for taking part in the POSEIDON study and our student employees and interns for their support with data acquisition and data entry.

# References

1. Boyd A, Golding J, Macleod J, Lawlor DA, Fraser A, Henderson J, et al. Cohort Profile: the 'children of the 90s'--the index offspring of the Avon Longitudinal Study of Parents and Children. Int J Epidemiol. 2013;42(1):111-27.

2. Fraser A, Macdonald-Wallis C, Tilling K, Boyd A, Golding J, Davey Smith G, et al. Cohort Profile: the Avon Longitudinal Study of Parents and Children: ALSPAC mothers cohort. Int J Epidemiol. 2013;42(1):97-110.

3. Min JL, Hemani G, Davey Smith G, Relton C, Suderman M. Meffil: efficient normalization and analysis of very large DNA methylation datasets. Bioinformatics. 2018;34(23):3983-9.

4. Relton CL, Gaunt T, McArdle W, Ho K, Duggirala A, Shihab H, et al. Data Resource Profile: Accessible Resource for Integrated Epigenomic Studies (ARIES). Int J Epidemiol. 2015;44(4):1181-90.

5. Bakulski KM, Feinberg JI, Andrews SV, Yang J, Brown S, S LM, et al. DNA methylation of cord blood cell types: Applications for mixed cell birth studies. Epigenetics. 2016;11(5):354-62.

6. Leek JT, Johnson WE, Parker HS, Jaffe AE, Storey JD. The sva package for removing batch effects and other unwanted variation in high-throughput experiments. Bioinformatics. 2012;28(6):882-3.

7. Schisterman EF, Silver RM, Lesher LL, Faraggi D, Wactawski-Wende J, Townsend JM, et al. Preconception low-dose aspirin and pregnancy outcomes: results from the EAGeR randomised trial. Lancet. 2014;384(9937):29-36.

8. Yeung EH, Guan W, Mumford SL, Silver RM, Zhang C, Tsai MY, Schisterman EF. Measured maternal prepregnancy anthropometry and newborn DNA methylation. Epigenomics. 2019;11(2):187-98.

9. Aryee MJ, Jaffe AE, Corrada-Bravo H, Ladd-Acosta C, Feinberg AP, Hansen KD, Irizarry RA. Minfi: a flexible and comprehensive Bioconductor package for the analysis of Infinium DNA methylation microarrays. Bioinformatics. 2014;30(10):1363-9.

10. Gervin K, Salas LA, Bakulski KM, van Zelm MC, Koestler DC, Wiencke JK, et al. Systematic evaluation and validation of reference and library selection methods for deconvolution of cord blood DNA methylation data. Clin Epigenetics. 2019;11(1):125.

11. Kooijman MN, Kruithof CJ, van Duijn CM, Duijts L, Franco OH, van IMH, et al. The Generation R Study: design and cohort update 2017. Eur J Epidemiol. 2016;31(12):1243-64.

12. Lehne B, Drong AW, Loh M, Zhang W, Scott WR, Tan ST, et al. A coherent approach for analysis of the Illumina HumanMethylation450 BeadChip improves data quality and performance in epigenome-wide association studies. Genome Biol. 2015;16(1):37.

13. Gaillard R, Steegers EA, de Jongste JC, Hofman A, Jaddoe VW. Tracking of fetal growth characteristics during different trimesters and the risks of adverse birth outcomes. Int J Epidemiol. 2014;43(4):1140-53.

14. Guxens M, Ballester F, Espada M, Fernandez MF, Grimalt JO, Ibarluzea J, et al. Cohort Profile: the INMA--INfancia y Medio Ambiente--(Environment and Childhood) Project. Int J Epidemiol. 2012;41(4):930-40.

15. van Iterson M, Tobi EW, Slieker RC, den Hollander W, Luijk R, Slagboom PE, Heijmans BT. MethylAid: visual and interactive quality control of large Illumina 450k datasets. Bioinformatics. 2014;30(23):3435-7.

16. Fortin JP, Fertig E, Hansen K. shinyMethyl: interactive quality control of Illumina 450k DNA methylation arrays in R. F1000Res. 2014;3:175.

17. Westerway SC, Davison A, Cowell S. Ultrasonic fetal measurements: new Australian standards for the new millennium. Aust N Z J Obstet Gynaecol. 2000;40(3):297-302.

18. Houseman EA, Accomando WP, Koestler DC, Christensen BC, Marsit CJ, Nelson HH, et al. DNA methylation arrays as surrogate measures of cell mixture distribution. BMC Bioinformatics. 2012;13:86.

19. Johnson WE, Li C, Rabinovic A. Adjusting batch effects in microarray expression data using empirical Bayes methods. Biostatistics. 2007;8(1):118-27.

20. Magnus P, Birke C, Vejrup K, Haugan A, Alsaker E, Daltveit AK, et al. Cohort Profile Update: The Norwegian Mother and Child Cohort Study (MoBa). Int J Epidemiol. 2016;45(2):382-8.

21. Ronningen KS, Paltiel L, Meltzer HM, Nordhagen R, Lie KK, Hovengen R, et al. The biobank of the Norwegian Mother and Child Cohort Study: a resource for the next 100 years. Eur J Epidemiol. 2006;21(8):619-25.

22. Magnus P, Irgens LM, Haug K, Nystad W, Skjaerven R, Stoltenberg C, MoBa Study G. Cohort profile: the Norwegian Mother and Child Cohort Study (MoBa). Int J Epidemiol. 2006;35(5):1146-50.

23. Haberg SE, London SJ, Nafstad P, Nilsen RM, Ueland PM, Vollset SE, Nystad W. Maternal folate levels in pregnancy and asthma in children at age 3 years. J Allergy Clin Immunol. 2011;127(1):262-4, 4 e1.

24. Joubert BR, den Dekker HT, Felix JF, Bohlin J, Ligthart S, Beckett E, et al. Maternal plasma folate impacts differential DNA methylation in an epigenome-wide meta-analysis of newborns. Nat Commun. 2016;7:10577.

25. Joubert BR, Haberg SE, Nilsen RM, Wang X, Vollset SE, Murphy SK, et al. 450K epigenome-wide scan identifies differential DNA methylation in newborns related to maternal smoking during pregnancy. Environ Health Perspect. 2012;120(10):1425-31.

26. Joubert BR, Felix JF, Yousefi P, Bakulski KM, Just AC, Breton C, et al. DNA Methylation in Newborns and Maternal Smoking in Pregnancy: Genome-wide Consortium Meta-analysis. Am J Hum Genet. 2016;98(4):680-96.

27. Bibikova M, Barnes B, Tsan C, Ho V, Klotzle B, Le JM, et al. High density DNA methylation array with single CpG site resolution. Genomics. 2011;98(4):288-95.

28. Teschendorff AE, Marabita F, Lechner M, Bartlett T, Tegner J, Gomez-Cabrero D, Beck S. A beta-mixture quantile normalization method for correcting probe design bias in Illumina Infinium 450 k DNA methylation data. Bioinformatics. 2013;29(2):189-96.

29. Witt SH, Frank J, Gilles M, Lang M, Treutlein J, Streit F, et al. Impact on birth weight of maternal smoking throughout pregnancy mediated by DNA methylation. BMC Genomics. 2018;19(1):290.

30. Send TS, Gilles M, Codd V, Wolf I, Bardtke S, Streit F, et al. Telomere Length in Newborns is Related to Maternal Stress During Pregnancy. Neuropsychopharmacology. 2017;42(12):2407-13.
