# Supplementary material for: Associations of maternal night shift work during pregnancy with DNA methylation in offspring: a meta-analysis in the PACE consortium
Source: Clin Epigenetics. 2025 Jan 22;17:12. doi: 10.1186/s13148-024-01810-y (PMC11756212; doi:10.1186/s13148-024-01810-y)
Supplement: Supplementary file 3 — Additional file3 (DOCX 2782 KB) [file 13148_2024_1810_MOESM3_ESM.docx]

## Supplementary Tables and Figures

## Supplementary Tables

**Supplementary Table 1 -** Gene ontology (GO) and Kyoto Encyclopedia of Genes and Genomes (KEGG) results with nominal P < 0.01

based on the 118 CpGs with P < 1×10^−4^ from the main model

| **ONTOLOGY** | **TERM** | **N** | **DE** | ***P* value** | **FDR** |
| --- | --- | --- | --- | --- | --- |
| GO BP | MHC protein complex assembly | 9 | 2 | 0.000967 | 1 |
| GO BP | peptide antigen assembly with MHC protein complex | 9 | 2 | 0.000967 | 1 |
| GO BP | positive regulation of transforming growth factor beta receptor signaling pathway | 31 | 3 | 0.001031 | 1 |
| GO BP | positive regulation of cellular response to transforming growth factor beta stimulus | 31 | 3 | 0.001031 | 1 |
| GO BP | smoothened signaling pathway | 134 | 5 | 0.002087 | 1 |
| GO BP | regulation of smoothened signaling pathway | 79 | 4 | 0.002224 | 1 |
| GO BP | prostate gland development | 44 | 3 | 0.003617 | 1 |
| GO BP | regulation of exoribonuclease activity | 1 | 1 | 0.004218 | 1 |
| GO BP | positive regulation of exoribonuclease activity | 1 | 1 | 0.004218 | 1 |
| GO BP | positive regulation of exonuclease activity | 1 | 1 | 0.004218 | 1 |
| GO BP | aspartate family amino acid biosynthetic process | 21 | 2 | 0.005177 | 1 |
| GO BP | Okazaki fragment processing involved in mitotic DNA replication | 1 | 1 | 0.005327 | 1 |
| GO BP | regulation of histone methylation | 62 | 3 | 0.005886 | 1 |
| GO BP | S-adenosylhomocysteine catabolic process | 1 | 1 | 0.008424 | 1 |
| GO BP | regulation of iron ion transmembrane transporter activity | 1 | 1 | 0.008562 | 1 |
| GO BP | positive regulation of iron ion transmembrane transporter activity | 1 | 1 | 0.008562 | 1 |
| GO BP | cellular modified amino acid catabolic process | 26 | 2 | 0.008679 | 1 |
| GO BP | myeloid dendritic cell antigen processing and presentation | 1 | 1 | 0.009082 | 1 |
| GO BP | antigen processing and presentation of endogenous peptide antigen via MHC class II | 1 | 1 | 0.009082 | 1 |
| GO BP | phytanic acid metabolic process | 2 | 1 | 0.009086 | 1 |
| GO BP | regulation of molecular function, epigenetic | 2 | 1 | 0.009315 | 1 |
| GO CC | integral component of lumenal side of endoplasmic reticulum membrane | 19 | 2 | 0.003842 | 1 |
| GO CC | lumenal side of endoplasmic reticulum membrane | 19 | 2 | 0.003842 | 1 |
| GO CC | lumenal side of membrane | 26 | 2 | 0.007086 | 1 |
| GO MF | preprotein binding | 1 | 1 | 0.003837 | 1 |
| GO MF | inositol-1,4-bisphosphate 1-phosphatase activity | 1 | 1 | 0.004486 | 1 |
| GO MF | betaine-homocysteine S-methyltransferase activity | 1 | 1 | 0.005013 | 1 |
| GO MF | hydroxypyruvate isomerase activity | 1 | 1 | 0.006006 | 1 |
| GO MF | adenosylhomocysteinase activity | 1 | 1 | 0.008424 | 1 |
| GO MF | trialkylsulfonium hydrolase activity | 1 | 1 | 0.008424 | 1 |
| KEGG | Cysteine and methionine metabolism | 49 | 3 | 0.002156 | 0.76 |

BP: Biologic Processes; CC: Cellular Component; MF: Molecular Function; N: number of genes in the GO or KEGG term;

DE: number of genes in the GO or KEGG term that are differentially methylated; FDR: false discovery rate-adjusted pvalue.

**Supplementary Table 2** - Examination of the placenta FDR hits in our cord blood results

| **Placenta FDR-hits** | **Effect Size** | **SE** | ***P* value** |
| --- | --- | --- | --- |
| **cg20296990 (-)** | **0.0019** | **0.0006** | **0.0023** |
| **cg11983245 (-)** | **0.0019** | **0.0009** | **0.0290** |
| **cg23601374 (-)** | **0.0025** | **0.0012** | **0.0312** |
| *** cg21373996 (-)** | **0.0015** | **0.0007** | **0.0347** |
| **cg16337763 (-)** | **0.0006** | **0.0003** | **0.0493** |
| *** cg14814323 (-)** | 0.0017 | 0.0009 | 0.0558 |
| **cg07425109 (-)** | 0.0033 | 0.0019 | 0.0801 |
| **cg23147227 (-)** | 0.0016 | 0.0009 | 0.0857 |
| **cg00625783 (-)** | -0.0027 | 0.0016 | 0.0930 |
| **cg18539461 (-)** | -0.0007 | 0.0004 | 0.1331 |
| **cg02530407 (-)** | 0.0015 | 0.0010 | 0.1366 |
| **cg04070692 (-)** | 0.0015 | 0.0010 | 0.1392 |
| **cg13877974 (-)** | -0.0006 | 0.0004 | 0.1431 |
| **cg01784220 (-)** | 0.0016 | 0.0011 | 0.1433 |
| **cg08174792 (-)** | -0.0020 | 0.0014 | 0.1579 |
| **cg10492999 (-)** | 0.0006 | 0.0005 | 0.1642 |
| *** cg14858786 (-)** | 0.0004 | 0.0003 | 0.1698 |
| **cg07618409 (-)** | 0.0011 | 0.0008 | 0.1778 |
| **cg05919312 (-)** | -0.0012 | 0.0009 | 0.1867 |
| **cg23289545 (-)** | 0.0009 | 0.0007 | 0.1888 |
| *** cg08082763 (-)** | 0.0010 | 0.0009 | 0.2510 |
| **cg03700230 (+)** | -0.0024 | 0.0022 | 0.2838 |
| **cg07225641 (-)** | -0.0023 | 0.0022 | 0.2938 |
| **cg04063235 (-)** | 0.0014 | 0.0014 | 0.3146 |
| **cg13626866 (-)** | 0.0012 | 0.0013 | 0.3433 |
| **cg13982098 (-)** | -0.0005 | 0.0006 | 0.3456 |
| **cg00080706 (-)** | -0.0009 | 0.0010 | 0.3547 |
| **cg12472449 (-)** | 0.0033 | 0.0041 | 0.4197 |
| **cg17985854 (-)** | 0.0014 | 0.0018 | 0.4307 |
| **cg06575572 (-)** | 0.0017 | 0.0023 | 0.4562 |
| **cg01022370 (-)** | -0.0010 | 0.0014 | 0.4901 |
| **cg06866814 (+)** | 0.0001 | 0.0002 | 0.5216 |
| *** cg06667732 (-)** | 0.0017 | 0.0027 | 0.5220 |
| **cg23181580 (-)** | -0.0006 | 0.0011 | 0.5707 |
| **cg05333740 (-)** | 0.0006 | 0.0011 | 0.5720 |
| **cg19906741 (+)** | 0.0004 | 0.0008 | 0.6000 |
| **cg24373865 (-)** | 0.0006 | 0.0012 | 0.6077 |
| **cg01422243 (-)** | 0.0007 | 0.0014 | 0.6120 |
| *** cg25342875 (-)** | 0.0005 | 0.0011 | 0.6200 |
| **cg00040588 (-)** | -0.0008 | 0.0017 | 0.6512 |
| **cg00762738 (-)** | -0.0002 | 0.0006 | 0.6782 |
| **cg13536107 (-)** | 0.0004 | 0.0011 | 0.6834 |
| **cg11221200 (-)** | -0.0003 | 0.0007 | 0.6880 |
| **cg21333033 (-)** | 0.0003 | 0.0007 | 0.7013 |
| **cg00168835 (+)** | -0.0001 | 0.0003 | 0.7063 |
| **cg04365973 (-)** | 0.0004 | 0.0010 | 0.7154 |
| *** cg14377596 (-)** | 0.0000 | 0.0001 | 0.7154 |
| **cg14168733 (-)** | 0.0007 | 0.0021 | 0.7440 |
| **cg16713168 (-)** | -0.0001 | 0.0007 | 0.8557 |
| *** cg01411786 (-)** | 0.0000 | 0.0002 | 0.8627 |
| *** cg18024167 (-)** | 0.0003 | 0.0019 | 0.8866 |

(+)/(-): direction of the association in placenta study; SE: standard error; *: CpG that met Bonferroni significance threshold in placenta tissue EWAS.

Bolded values correspond to nominal significance in the cord blood study.

**Supplementary Table 3** - Examination of the FDR-significant hits in the childhood mQTL database for genetic effects on DNA methylation

| **Timepoint** | **SNP** | **SNP Chr** | **SNP Pos** | **A1** | **A2** | **MAF** | **CpG** | **CpG Chr** | **CpG Pos** | **Effect Size** | **p-value** | ***Cis/Trans*** |
| --- | --- | --- | --- | --- | --- | --- | --- | --- | --- | --- | --- | --- |
| Pregnancy | rs4655563 | 1 | 68019721 | C | T | 0.407 | cg00773359 | 7 | 805214 | 0.0037 | 9.76×10^-08^ | *trans* |
| Pregnancy | rs2299377 | 7 | 103373014 | A | G | 0.417 | cg10945885 | 1 | 22665067 | 0.0089 | 2.31×10^-08^ | *trans* |
| Pregnancy | rs2299378 | 7 | 103373033 | C | T | 0.499 | cg10945885 | 1 | 22665067 | 0.0090 | 1.84×10^-08^ | *trans* |
| Pregnancy | rs2299379 | 7 | 103373051 | T | C | 0.41 | cg10945885 | 1 | 22665067 | 0.0088 | 7.04×10^-08^ | *trans* |
| Pregnancy | rs2299380 | 7 | 103373086 | T | G | 0.498 | cg10945885 | 1 | 22665067 | 0.0090 | 2.11×10^-08^ | *trans* |
| Adolescence | rs4413993 | 1 | 233935193 | T | C | 0.291 | cg10945885 | 1 | 22665067 | 0.0089 | 8.27×10^-08^ | *trans* |
| Pregnancy | rs671372 | 7 | 103372911 | G | T | 0.498 | cg10945885 | 1 | 22665067 | 0.0086 | 2.77×10^-08^ | *trans* |
| Birth | rs117371715 | 10 | 118632963 | C | T | 0.01 | cg21836426 | 4 | 184580106 | 0.0000 | 8.27×10^-09^ | *trans* |
| Middle Age | rs141587506 | 10 | 53214307 | A | G | 0.014 | cg21836426 | 4 | 184580106 | 0.0000 | 2.45×10^-08^ | *trans* |
| Adolescence | rs142140959 | 23 | 96310327 | G | C | 0.007 | cg21836426 | 4 | 184580106 | 0.0042 | 3.65×10^-09^ | *trans* |
| Middle Age | rs150886189 | 10 | 53215034 | G | A | 0.014 | cg21836426 | 4 | 184580106 | 0.0000 | 2.45×10^-08^ | *trans* |
| Birth | rs183291851 | 10 | 118774349 | T | G | 0.01 | cg21836426 | 4 | 184580106 | 0.0000 | 6.61×10^-08^ | *trans* |
| Adolescence | rs183905669 | X | 95891531 | C | T | 0.006 | cg21836426 | 4 | 184580106 | 0.0042 | 1.89×10^-09^ | *trans* |
| Adolescence | rs184633165 | X | 95844804 | T | C | 0.005 | cg21836426 | 4 | 184580106 | 0.0042 | 9.86×10^-10^ | *trans* |
| Adolescence | rs186076958 | X | 96355096 | G | A | 0.007 | cg21836426 | 4 | 184580106 | 0.0042 | 3.85×10^-08^ | *trans* |
| Pregnancy | rs62193427 | 20 | 24344783 | T | C | 0.008 | cg21836426 | 4 | 184580106 | 0.0000 | 4.49×10^-08^ | *trans* |

SNP: Single nucleotide polymorphism; Chr: chromosome; Pos: position; A: allele; MAF: Minor allele frequency

**Supplementary Table 4** - Examination of the FDR-significant hits in the GoDMC mQTL database for genetic effects on DNA methylation

| **SNP** | **SNP Chr** | **SNP Pos** | **A1** | **A2** | **CpG** | **CpG Chr** | **CpG Pos** | **Effect Size** | **p-value** | ***Cis/Trans*** |
| --- | --- | --- | --- | --- | --- | --- | --- | --- | --- | --- |
| rs10917216 | 1 | 22665400 | C | A | cg10945885 | 1 | 22665067 | 0.1362 | 3.37×10^-52^ | *cis* |
| rs10917217 | 1 | 22669472 | A | C | cg10945885 | 1 | 22665067 | 0.1345 | 2.12×10^-47^ | *cis* |
| rs1343987 | 1 | 22679309 | T | C | cg10945885 | 1 | 22665067 | -0.1040 | 3.14×10^-19^ | *cis* |
| rs1934477 | 1 | 22664774 | T | C | cg10945885 | 1 | 22665067 | 0.1359 | 4.38×10^-49^ | *cis* |
| rs1934478 | 1 | 22665210 | G | A | cg10945885 | 1 | 22665067 | 0.1367 | 2.38×10^-52^ | *cis* |
| rs4233285 | 1 | 22676292 | T | C | cg10945885 | 1 | 22665067 | 0.1257 | 8.97×10^-41^ | *cis* |
| rs4655043 | 1 | 22654254 | A | G | cg10945885 | 1 | 22665067 | 0.1184 | 1.24×10^-39^ | *cis* |
| rs4655044 | 1 | 22659375 | C | T | cg10945885 | 1 | 22665067 | 0.1358 | 4.83×10^-50^ | *cis* |
| rs7553482 | 1 | 22669656 | G | A | cg10945885 | 1 | 22665067 | 0.1254 | 1.59×10^-43^ | *cis* |

SNP: Single nucleotide polymorphism; Chr: chromosome; Pos: position; A: allele

**Supplementary Table 5 -** Examination of the FDR-significant hits in the blood autosomal cis-eQTM catalogue from the Human Early Life Exposome (HELIX) project

| **CpG** | **CpG**  **chr** | **CpG**  **pos** | **CpG**  **gene** | **TC** | **TC gene**  **start** | **TC gene**  **end** | **TC gene**  **TSS** | **TC gene** | **log2FC** | **SE** | ***P* value** | **sigPair** |
| --- | --- | --- | --- | --- | --- | --- | --- | --- | --- | --- | --- | --- |
| cg10945885 | chr1 | 22665067 |  | TC01000278.hg.1 | 22337478 | 22337581 | 22337478 |  | 0.0947 | 0.5435 | 0.0817 | FALSE |
| cg10945885 | chr1 | 22665067 |  | TC01002328.hg.1 | 22148737 | 22263790 | 22263790 | *HSPG2* | 0.0113 | 0.0686 | 0.0995 | FALSE |
| cg10945885 | chr1 | 22665067 |  | TC01004236.hg.1 | 22582820 | 22584588 | 22582820 |  | -0.0436 | 0.2877 | 0.1301 | FALSE |
| cg10945885 | chr1 | 22665067 |  | TC01000286.hg.1 | 22970118 | 22974603 | 22970118 | *C1QC* | -0.0523 | 0.3945 | 0.1850 | FALSE |
| cg10945885 | chr1 | 22665067 |  | TC01000275.hg.1 | 22234678 | 22235073 | 22234678 |  | -0.0633 | 0.5058 | 0.2111 | FALSE |
| cg10945885 | chr1 | 22665067 |  | TC01000281.hg.1 | 22385690 | 22390692 | 22385690 |  | 0.0938 | 0.7514 | 0.2122 | FALSE |
| cg10945885 | chr1 | 22665067 |  | TC01000284.hg.1 | 22890004 | 22930087 | 22890004 | *EPHA8* | -0.0143 | 0.1187 | 0.2282 | FALSE |
| cg10945885 | chr1 | 22665067 |  | TC01004237.hg.1 | 23007533 | 23008941 | 23007533 |  | 0.0530 | 0.4754 | 0.2653 | FALSE |
| cg10945885 | chr1 | 22665067 |  | TC01005320.hg.1 | 22744726 | 22745435 | 22745435 |  | -0.0298 | 0.3165 | 0.3466 | FALSE |
| cg10945885 | chr1 | 22665067 |  | TC01000290.hg.1 | 23075310 | 23075404 | 23075310 |  | 0.0620 | 0.7089 | 0.3821 | FALSE |
| cg10945885 | chr1 | 22665067 |  | TC01000288.hg.1 | 23037331 | 23241823 | 23037331 | *EPHB2* | 0.0163 | 0.1927 | 0.3976 | FALSE |
| cg10945885 | chr1 | 22665067 |  | TC01004234.hg.1 | 22224268 | 22230303 | 22224268 |  | -0.0201 | 0.2583 | 0.4356 | FALSE |
| cg10945885 | chr1 | 22665067 |  | TC01000283.hg.1 | 22843967 | 22846201 | 22843967 |  | -0.0387 | 0.5449 | 0.4774 | FALSE |
| cg10945885 | chr1 | 22665067 |  | TC01005317.hg.1 | 22422079 | 22431542 | 22431542 |  | 0.0278 | 0.4126 | 0.5003 | FALSE |
| cg10945885 | chr1 | 22665067 |  | TC01000280.hg.1 | 22379120 | 22419437 | 22379120 | *CDC42* | -0.0119 | 0.2245 | 0.5950 | FALSE |
| cg10945885 | chr1 | 22665067 |  | TC01000279.hg.1 | 22351681 | 22357716 | 22351681 | *LINC00339* | 0.0357 | 0.9220 | 0.6986 | FALSE |
| cg10945885 | chr1 | 22665067 |  | TC01002329.hg.1 | 22350487 | 22351461 | 22351461 |  | 0.0126 | 0.3300 | 0.7024 | FALSE |
| cg10945885 | chr1 | 22665067 |  | TC01000287.hg.1 | 22979255 | 22988031 | 22979255 | *C1QB* | -0.0106 | 0.2838 | 0.7077 | FALSE |
| cg10945885 | chr1 | 22665067 |  | TC01005319.hg.1 | 22691123 | 22692975 | 22692975 |  | 0.0121 | 0.3913 | 0.7575 | FALSE |
| cg10945885 | chr1 | 22665067 |  | TC01005315.hg.1 | 22223074 | 22224048 | 22224048 |  | 0.0078 | 0.3816 | 0.8381 | FALSE |
| cg10945885 | chr1 | 22665067 |  | TC01000282.hg.1 | 22778344 | 22857650 | 22778344 | *ZBTB40* | 0.0061 | 0.3172 | 0.8466 | FALSE |
| cg10945885 | chr1 | 22665067 |  | TC01004235.hg.1 | 22351707 | 22357713 | 22351707 |  | -0.0117 | 0.6243 | 0.8512 | FALSE |
| cg10945885 | chr1 | 22665067 |  | TC01000285.hg.1 | 22962999 | 22966175 | 22962999 | *C1QA* | -0.0023 | 0.1938 | 0.9041 | FALSE |
| cg10945885 | chr1 | 22665067 |  | TC01005316.hg.1 | 22350487 | 22357707 | 22357707 | *LOC101928043* | -0.0017 | 0.1906 | 0.9306 | FALSE |
| cg10945885 | chr1 | 22665067 |  | TC01006313.hg.1 | 22303418 | 22325135 | 22303418 | *CELA3B* | 0.0011 | 0.1422 | 0.9356 | FALSE |
| cg10945885 | chr1 | 22665067 |  | TC01002331.hg.1 | 23162206 | 23163342 | 23163342 |  | 0.0021 | 0.4357 | 0.9622 | FALSE |
| cg10945885 | chr1 | 22665067 |  | TC01002330.hg.1 | 22443798 | 22470462 | 22470462 | *WNT4* | 0.0000 | 0.1568 | 0.9991 | FALSE |
| cg21836426 | chr4 | 184580106 | *RWDD4A*  *C4orf41* | TC04002870.hg.1 | 1.85E+08 | 1.85E+08 | 1.85E+08 |  | 0.1086 | 0.6694 | 0.10516 | FALSE |
| cg21836426 | chr4 | 184580106 | *RWDD4A**  *C4orf41** | TC04000891.hg.1 | 1.85E+08 | 1.85E+08 | 1.85E+08 | *TRAPPC11** | 0.0709 | 0.4468 | 0.11292 | FALSE |
| cg21836426 | chr4 | 184580106 | *RWDD4A*  *C4orf41* | TC04002354.hg.1 | 1.85E+08 | 1.85E+08 | 1.85E+08 |  | -0.1007 | 0.6901 | 0.14489 | FALSE |
| cg21836426 | chr4 | 184580106 | *RWDD4A*  *C4orf41* | TC04002865.hg.1 | 1.84E+08 | 1.84E+08 | 1.84E+08 | *WWC2-AS1* | -0.1178 | 0.8319 | 0.15720 | FALSE |
| cg21836426 | chr4 | 184580106 | *RWDD4A*  *C4orf41* | TC04002866.hg.1 | 1.84E+08 | 1.84E+08 | 1.84E+08 |  | -0.0800 | 0.5939 | 0.17856 | FALSE |
| cg21836426 | chr4 | 184580106 | *RWDD4A*  *C4orf41* | TC04001791.hg.1 | 1.84E+08 | 1.84E+08 | 1.84E+08 | *WWC2-AS1* | -0.1013 | 0.7993 | 0.20562 | FALSE |
| cg21836426 | chr4 | 184580106 | *RWDD4A**  *C4orf41** | TC04001797.hg.1 | 1.85E+08 | 1.85E+08 | 1.85E+08 | *RWDD4** | -0.0494 | 0.5130 | 0.33609 | FALSE |
| cg21836426 | chr4 | 184580106 | *RWDD4A*  *C4orf41* | TC04000889.hg.1 | 1.84E+08 | 1.84E+08 | 1.84E+08 | *CDKN2AIP* | 0.0454 | 0.4798 | 0.34451 | FALSE |
| cg21836426 | chr4 | 184580106 | *RWDD4A*  *C4orf41* | TC04000893.hg.1 | 1.85E+08 | 1.85E+08 | 1.85E+08 | *STOX2* | 0.0310 | 0.3537 | 0.38085 | FALSE |
| cg21836426 | chr4 | 184580106 | *RWDD4A*  *C4orf41* | TC04002351.hg.1 | 1.84E+08 | 1.84E+08 | 1.84E+08 |  | -0.0813 | 0.9709 | 0.40264 | FALSE |
| cg21836426 | chr4 | 184580106 | *RWDD4A*  *C4orf41* | TC04001795.hg.1 | 1.84E+08 | 1.84E+08 | 1.84E+08 | *LOC389247* | -0.0593 | 0.7103 | 0.40385 | FALSE |
| cg21836426 | chr4 | 184580106 | *RWDD4A*  *C4orf41* | TC04002869.hg.1 | 1.84E+08 | 1.84E+08 | 1.84E+08 |  | -0.0552 | 0.7253 | 0.44684 | FALSE |
| cg21836426 | chr4 | 184580106 | *RWDD4A*  *C4orf41* | TC04001789.hg.1 | 1.84E+08 | 1.84E+08 | 1.84E+08 |  | -0.0819 | 1.1134 | 0.46221 | FALSE |
| cg21836426 | chr4 | 184580106 | *RWDD4A*  *C4orf41* | TC04002353.hg.1 | 1.85E+08 | 1.85E+08 | 1.85E+08 |  | 0.0390 | 0.5340 | 0.46505 | FALSE |
| cg21836426 | chr4 | 184580106 | *RWDD4A*  *C4orf41* | TC04001790.hg.1 | 1.84E+08 | 1.84E+08 | 1.84E+08 |  | 0.0541 | 0.7651 | 0.47936 | FALSE |
| cg21836426 | chr4 | 184580106 | *RWDD4A*  *C4orf41* | TC04000895.hg.1 | 1.85E+08 | 1.85E+08 | 1.85E+08 | *STOX2* | 0.0466 | 0.6776 | 0.49194 | FALSE |
| cg21836426 | chr4 | 184580106 | *RWDD4A*  *C4orf41* | TC04002356.hg.1 | 1.85E+08 | 1.85E+08 | 1.85E+08 | *STOX2* | 0.0318 | 0.6556 | 0.62753 | FALSE |
| cg21836426 | chr4 | 184580106 | *RWDD4A*  *C4orf41* | TC04000890.hg.1 | 1.84E+08 | 1.84E+08 | 1.84E+08 | *ING2* | -0.0236 | 0.5426 | 0.66338 | FALSE |
| cg21836426 | chr4 | 184580106 | *RWDD4A*  *C4orf41* | TC04001799.hg.1 | 1.85E+08 | 1.85E+08 | 1.85E+08 |  | -0.0427 | 0.9871 | 0.66513 | FALSE |
| cg21836426 | chr4 | 184580106 | *RWDD4A*  *C4orf41* | TC04001793.hg.1 | 1.84E+08 | 1.84E+08 | 1.84E+08 | *CLDN22* | -0.0373 | 0.9875 | 0.70578 | FALSE |
| cg21836426 | chr4 | 184580106 | *RWDD4A*  *C4orf41* | TC04002355.hg.1 | 1.85E+08 | 1.85E+08 | 1.85E+08 |  | 0.0344 | 0.9604 | 0.72001 | FALSE |
| cg21836426 | chr4 | 184580106 | *RWDD4A*  *C4orf41* | TC04002352.hg.1 | 1.85E+08 | 1.85E+08 | 1.85E+08 |  | 0.0246 | 0.6941 | 0.72282 | FALSE |
| cg21836426 | chr4 | 184580106 | *RWDD4A*  *C4orf41* | TC04002868.hg.1 | 1.84E+08 | 1.84E+08 | 1.84E+08 |  | 0.0303 | 0.9176 | 0.74140 | FALSE |
| cg21836426 | chr4 | 184580106 | *RWDD4A*  *C4orf41* | TC04000888.hg.1 | 1.84E+08 | 1.84E+08 | 1.84E+08 |  | 0.0287 | 0.9814 | 0.76981 | FALSE |
| cg21836426 | chr4 | 184580106 | *RWDD4A*  *C4orf41* | TC04001792.hg.1 | 1.84E+08 | 1.84E+08 | 1.84E+08 |  | -0.0206 | 0.9427 | 0.82686 | FALSE |
| cg21836426 | chr4 | 184580106 | *RWDD4A*  *C4orf41* | TC04001796.hg.1 | 1.85E+08 | 1.85E+08 | 1.85E+08 |  | -0.0238 | 1.3212 | 0.85701 | FALSE |
| cg21836426 | chr4 | 184580106 | *RWDD4A*  *C4orf41* | TC04000894.hg.1 | 1.85E+08 | 1.85E+08 | 1.85E+08 |  | 0.0054 | 0.6612 | 0.93449 | FALSE |
| cg21836426 | chr4 | 184580106 | *RWDD4A*  *C4orf41* | TC04001798.hg.1 | 1.85E+08 | 1.85E+08 | 1.85E+08 |  | -0.0068 | 1.3125 | 0.95874 | FALSE |
| cg21836426 | chr4 | 184580106 | *RWDD4A*  *C4orf41* | TC04002350.hg.1 | 1.84E+08 | 1.84E+08 | 1.84E+08 |  | 0.0026 | 0.8012 | 0.97456 | FALSE |
| cg21836426 | chr4 | 184580106 | *RWDD4A*  *C4orf41* | TC04000892.hg.1 | 1.85E+08 | 1.85E+08 | 1.85E+08 |  | -0.0038 | 1.3105 | 0.97691 | FALSE |
| **cg00773359** | **chr7** | **805214** | ***HEATR2**** | **TC07000011.hg.1** | **766338** | **829190** | **766338** | ***DNAAF5**** | **-0.0909** | **0.2601** | **0.00050** | **FALSE** |
| **cg00773359** | **chr7** | **805214** | ***HEATR2*** | **TC07002094.hg.1** | **419325** | **422845** | **419325** | ***LOC442497*** | **0.1522** | **0.5487** | **0.00565** | **FALSE** |
| **cg00773359** | **chr7** | **805214** | ***HEATR2*** | **TC07000010.hg.1** | **642822** | **648119** | **642822** |  | **-0.1496** | **0.7066** | **0.03455** | **FALSE** |
| **cg00773359** | **chr7** | **805214** | ***HEATR2*** | **TC07001081.hg.1** | **1193840** | **1196333** | **1196333** | ***ZFAND2A*** | **-0.1331** | **0.6539** | **0.04215** | **FALSE** |
| cg00773359 | chr7 | 805214 | *HEATR2* | TC07003315.hg.1 | 855528 | 914557 | 855528 | *SUN1* | -0.0560 | 0.2939 | 0.05732 | FALSE |
| cg00773359 | chr7 | 805214 | *HEATR2* | TC07000014.hg.1 | 1068661 | 1083527 | 1068661 |  | 0.1079 | 0.6263 | 0.08520 | FALSE |
| cg00773359 | chr7 | 805214 | *HEATR2* | TC07002098.hg.1 | 1200128 | 1205594 | 1200128 | *LOC101927021* | -0.1092 | 0.6667 | 0.10172 | FALSE |
| cg00773359 | chr7 | 805214 | *HEATR2* | TC07001072.hg.1 | 523744 | 535755 | 535755 |  | -0.0877 | 0.5462 | 0.10856 | FALSE |
| cg00773359 | chr7 | 805214 | *HEATR2* | TC07000019.hg.1 | 1199662 | 1205594 | 1199662 | *LOC101927021* | -0.0564 | 0.3658 | 0.12346 | FALSE |
| cg00773359 | chr7 | 805214 | *HEATR2* | TC07000017.hg.1 | 1120499 | 1121814 | 1120499 |  | -0.0957 | 0.6449 | 0.13840 | FALSE |
| cg00773359 | chr7 | 805214 | *HEATR2* | TC07000006.hg.1 | 419325 | 422845 | 419325 | *LOC442497* | 0.0799 | 0.5623 | 0.15548 | FALSE |
| cg00773359 | chr7 | 805214 | *HEATR2* | TC07001073.hg.1 | 536895 | 559933 | 559933 | *PDGFA* | -0.0330 | 0.2942 | 0.26264 | FALSE |
| cg00773359 | chr7 | 805214 | *HEATR2* | TC07002095.hg.1 | 465456 | 468067 | 465456 |  | 0.0670 | 0.6442 | 0.29856 | FALSE |
| cg00773359 | chr7 | 805214 | *HEATR2* | TC07000020.hg.1 | 1272543 | 1276954 | 1272543 | *UNCX* | -0.0345 | 0.3662 | 0.34581 | FALSE |
| cg00773359 | chr7 | 805214 | *HEATR2* | TC07000009.hg.1 | 601595 | 605256 | 601595 |  | 0.0359 | 0.3884 | 0.35532 | FALSE |
| cg00773359 | chr7 | 805214 | *HEATR2* | TC07001080.hg.1 | 1191707 | 1200395 | 1200395 | *ZFAND2A* | 0.0527 | 0.6664 | 0.42945 | FALSE |
| cg00773359 | chr7 | 805214 | *HEATR2* | TC07000018.hg.1 | 1121844 | 1133451 | 1121844 | *GPER1* | 0.0243 | 0.3152 | 0.44153 | FALSE |
| cg00773359 | chr7 | 805214 | *HEATR2* | TC07003347.hg.1 | 1004486 | 1015235 | 1015235 | *COX19* | 0.0364 | 0.4742 | 0.44347 | FALSE |
| cg00773359 | chr7 | 805214 | *HEATR2* | TC07002742.hg.1 | 389824 | 390706 | 390706 |  | -0.1236 | 1.6194 | 0.44567 | FALSE |
| cg00773359 | chr7 | 805214 | *HEATR2* | TC07000016.hg.1 | 1114086 | 1117672 | 1114086 |  | -0.0616 | 0.8200 | 0.45245 | FALSE |
| cg00773359 | chr7 | 805214 | *HEATR2* | TC07002097.hg.1 | 1097330 | 1097781 | 1097330 | *GPR146* | -0.0774 | 1.0349 | 0.45501 | FALSE |
| cg00773359 | chr7 | 805214 | *HEATR2* | TC07000007.hg.1 | 560028 | 564869 | 560028 | *HRAT92* | -0.0320 | 0.6238 | 0.60828 | FALSE |
| cg00773359 | chr7 | 805214 | *HEATR2* | TC07003316.hg.1 | 916189 | 936073 | 916189 | *GET4* | -0.0150 | 0.2924 | 0.60856 | FALSE |
| cg00773359 | chr7 | 805214 | *HEATR2* | TC07002743.hg.1 | 490270 | 502281 | 502281 |  | -0.0336 | 0.6888 | 0.62546 | FALSE |
| cg00773359 | chr7 | 805214 | *HEATR2* | TC07001075.hg.1 | 756256 | 766614 | 766614 | *PRKAR1B* | -0.0287 | 0.6420 | 0.65490 | FALSE |
| cg00773359 | chr7 | 805214 | *HEATR2* | TC07001077.hg.1 | 1036623 | 1177896 | 1177896 | *C7orf50* | -0.0120 | 0.2960 | 0.68444 | FALSE |
| cg00773359 | chr7 | 805214 | *HEATR2* | TC07000013.hg.1 | 1022835 | 1029276 | 1022835 | *CYP2W1* | -0.0092 | 0.2386 | 0.69848 | FALSE |
| cg00773359 | chr7 | 805214 | *HEATR2* | TC07000005.hg.1 | 330136 | 334388 | 330136 | *WI2-2373I1.2* | -0.0172 | 0.4506 | 0.70256 | FALSE |
| cg00773359 | chr7 | 805214 | *HEATR2* | TC07001079.hg.1 | 1094996 | 1098897 | 1098897 |  | 0.0217 | 0.5954 | 0.71592 | FALSE |
| cg00773359 | chr7 | 805214 | *HEATR2* | TC07002099.hg.1 | 1273515 | 1275429 | 1273515 |  | 0.0201 | 0.5708 | 0.72461 | FALSE |
| cg00773359 | chr7 | 805214 | *HEATR2* | TC07002744.hg.1 | 1019650 | 1020276 | 1020276 |  | -0.0220 | 0.6425 | 0.73264 | FALSE |
| cg00773359 | chr7 | 805214 | *HEATR2* | TC07002093.hg.1 | 389257 | 391503 | 389257 |  | 0.0267 | 0.8419 | 0.75147 | FALSE |
| cg00773359 | chr7 | 805214 | *HEATR2* | TC07002096.hg.1 | 560028 | 565027 | 560028 |  | 0.0093 | 0.2957 | 0.75244 | FALSE |
| cg00773359 | chr7 | 805214 | *HEATR2* | TC07002745.hg.1 | 1064141 | 1067975 | 1067975 |  | 0.0060 | 0.4276 | 0.88861 | FALSE |
| cg00773359 | chr7 | 805214 | *HEATR2* | TC07001074.hg.1 | 588834 | 767313 | 767313 | *PRKAR1B* | 0.0026 | 0.2252 | 0.90971 | FALSE |
| cg00773359 | chr7 | 805214 | *HEATR2* | TC07003348.hg.1 | 937537 | 995043 | 995043 | *ADAP1* | 0.0021 | 0.2981 | 0.94295 | FALSE |
| cg00773359 | chr7 | 805214 | *HEATR2* | TC07002092.hg.1 | 331195 | 334385 | 331195 |  | -0.0040 | 0.6861 | 0.95375 | FALSE |
| cg00773359 | chr7 | 805214 | *HEATR2* | TC07000015.hg.1 | 1084212 | 1098897 | 1084212 | *GPR146* | 0.0007 | 0.3733 | 0.98522 | FALSE |

CpG: cytosine-phosphate-guanine site

CpG chr = CpG Chromosome

CpG pos = CpG position

TC gene start = start position of the Transcript Cluster (gene)

TC gene end = end position of the Transcript Cluster (gene)

TC gene TSS = position used as Transcription Start Site for the Transcript Cluster (gene)

TC gene = genes annotated to Transcript Cluster (gene) according to Affymetrix na36 annotation

log2FC = change in expression as log2 fold change per 10% DNA methylation

P value = P value of the CpG-TC association

sigPair = the pair significant after multiple testing correction (also for the number of TCs each CpG is paired with)? TRUE/FALS

* Alternative gene names:  *RWDD4A* = *RWDD4*; *C4orf41* = *TRAPPC11*; *HEATR2* = *DNAAF5*

**Supplementary Table 6 –** Examination of annotated genes to the 16 CpGs with P < 1×10^−5^ in the jet lag animal model

| Marker Name | Chr | Human  Gene | Mouse Gene | Chr | CpG count | DMR length | Fold Change | Validation^1^ |
| --- | --- | --- | --- | --- | --- | --- | --- | --- |
| cg10945885* | 1 | *ZBTB40* | *Zbtb40* | 4 | 19 | 1988 | 3.769 | Yes |
|  |  |  | *Zbtb40* | 4 | 16 | 1066 | 3.106 |  |
|  |  |  | *Zbtb40* | 4 | 29 | 1788 | 2.933 |  |
|  |  |  | *Zbtb40* | 4 | 18 | 1553 | 2.543 |  |
|  |  | *WNT4* | *Wnt4* | 4 | 49 | 2715 | 3.333 |  |
| cg21836426 | 4 | *RWDD4A*;  *C4orf41* | *Rwdd4a* | 8 | 12 | 1028 | 11.666 | Yes |
| cg00773359 | 7 | *HEATR2* | -- | -- | -- | -- | -- | No |
| cg25933594* | 4 | *FRG1* | -- | -- | -- | -- | -- | No |
| ch.14.955325R | 14 | *ACTN1* | *Actn1* | 12 | 31 | 2034 | 2.650 | Yes |
| cg01005536 | 16 | *RAB11FIP3* | *Rab11fip3* | 17 | 16 | 1032 | 2.911 | Yes |
|  |  |  | *Rab11fip3* | 17 | 22 | 1417 | 2.535 |  |
|  |  |  | *Rab11fip3* | 17 | 21 | 1880 | 5.162 |  |
| cg08437570 | 10 | *MIR146B* | -- | -- | -- | -- | -- | No |
| cg09670616 | 2 | *ZEB2* | *Zeb2* | 17 | 13 | 1613 | 6.963 | Yes |
| cg02311152 | 14 | *MAP3K9* | *Map3k9* | 12 | 27 | 1714 | 3.173 | Yes |
| cg23913995* | 20 | *NKX2-2* | near *Nkx2-2* | 2 | 35 | 1449 | 3.523 | No^2^ |
| cg04079538 | 1 | *TTC39A* | -- | -- | -- | -- | -- | No |
| cg05529152* | 12 | *GRASP* | *Grasp* | 15 | 81 | 3336 | 3.224 | Yes |
| cg22087659 | 7 | *TRIM4* | -- | -- | -- | -- | -- | No |
| cg23696886 | 8 | *PDLIM2* | -- | -- | -- | -- | -- | No |
| cg01039573 | 19 | *SHC2* | *Shc2* | 10 | 35 | 2234 | 2.809 | Yes |
| cg11973682 | 4 | *CRMP1* | -- | -- | -- | -- | -- | No |

* Human CpG not annotated to a gene by Illumina. Genes reported are the nearest genes to the CpG according to CSC Genome Browser.

^1^ Validation is “Yes” when we find a DMR in the mouse gene matching to the human gene and “No” otherwise. ^2^ DMR near the gene but not in the gene body.

Chr, chromosome; DMR, differently methylated region.

## Supplementary Figures


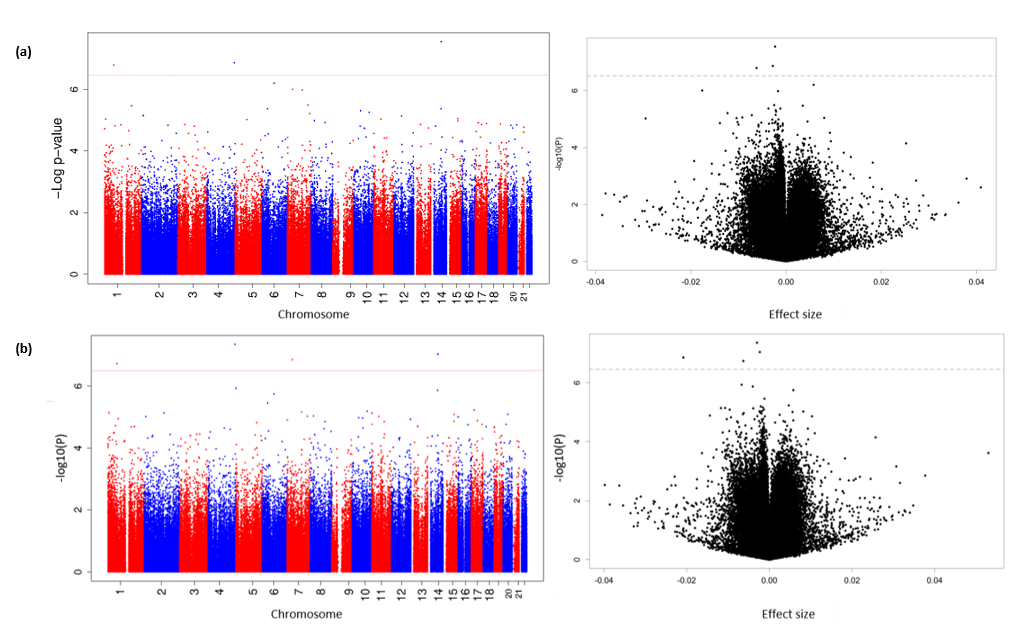


**Supplementary Figure 1 -** Manhattan and volcano plots of the crude (a) and reduced (b) main model meta-analysis of maternal shift work during pregnancy and offspring cord blood DNA methylation. Dashed lines indicate the cutoff for the adjusted p value adjusted for FDR at 5% significance level.


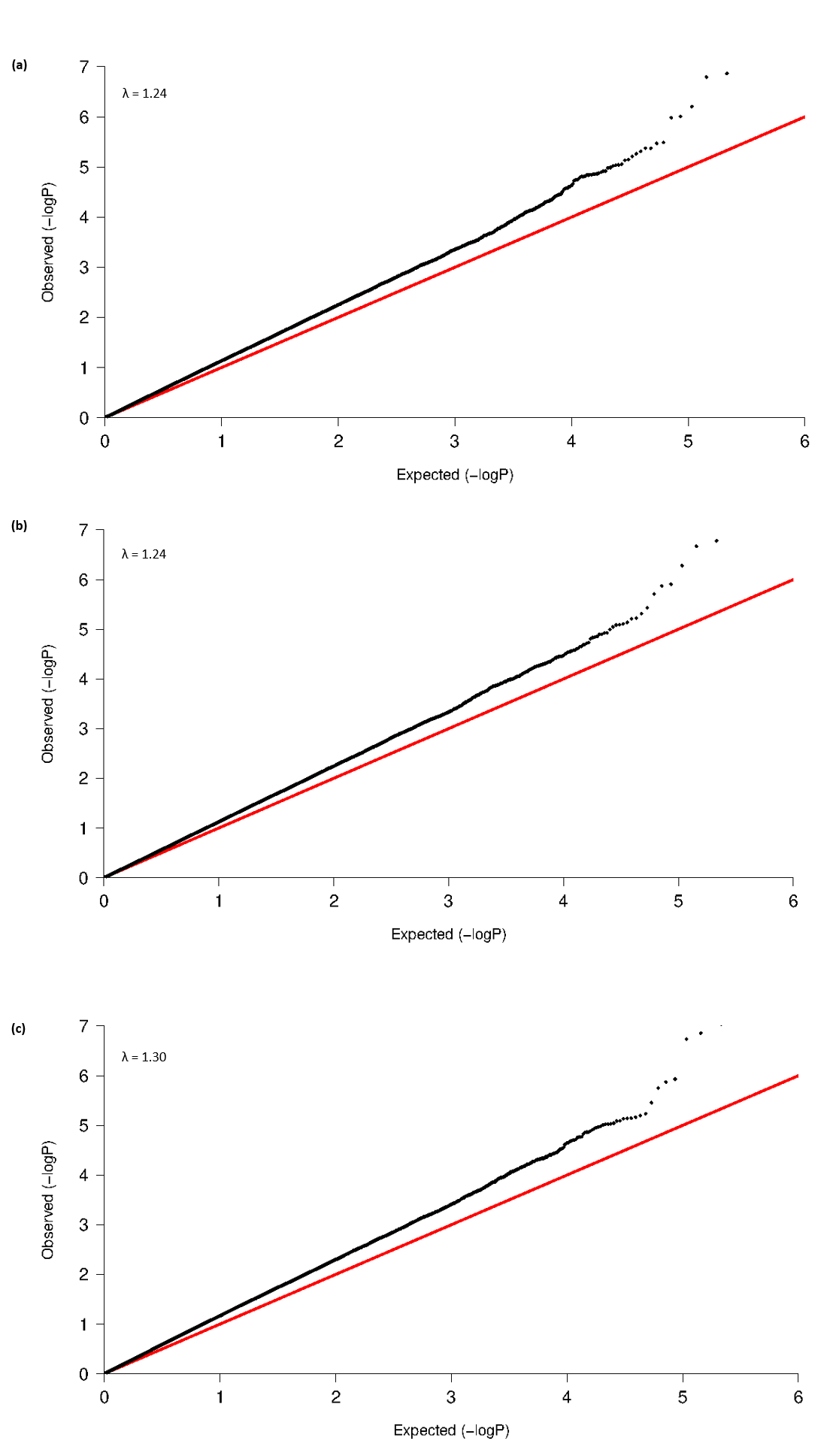


**Supplementary Figure 2 -** QQ plot and lambda (λ) values for crude (a), main (b) and reduced main (c) models


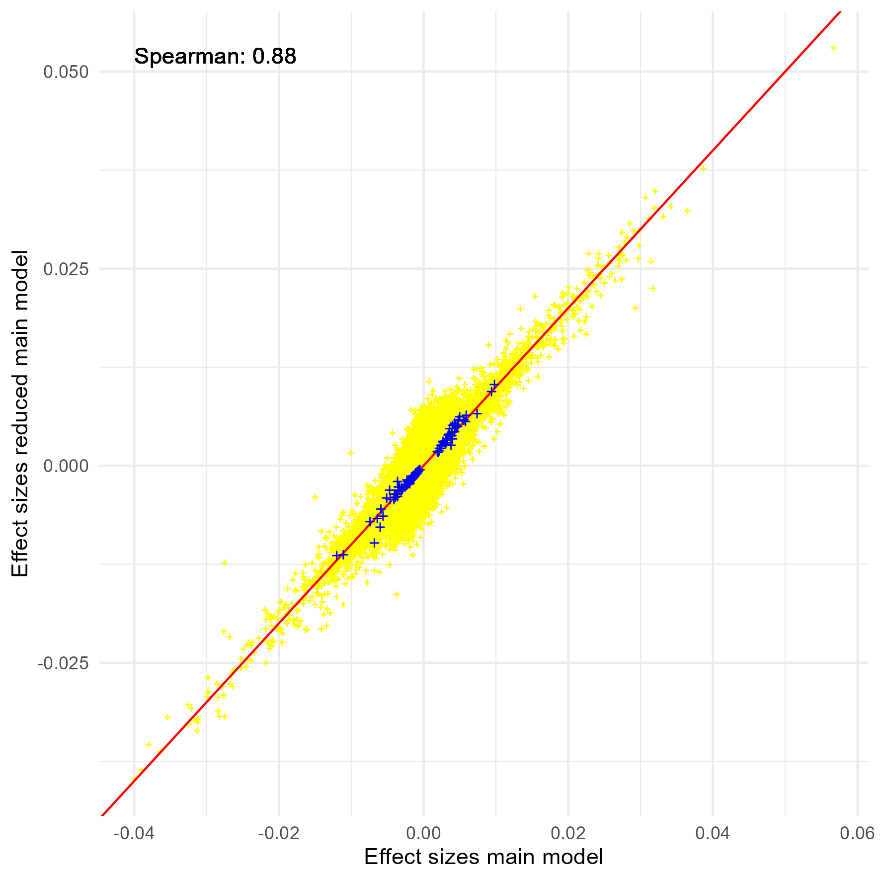


**Supplementary Figure 3 -** Effect sizes from the main models (X-axis) plotted against effect sizes of the reduced main model (excluding cell type composition from the covariates list, Y-axis). Y = X line is plotted in a red line. The 118 CpGs with nominal P value < 1×10^-4^ from the main model are colored blue, and all other CpGs are colored yellow.


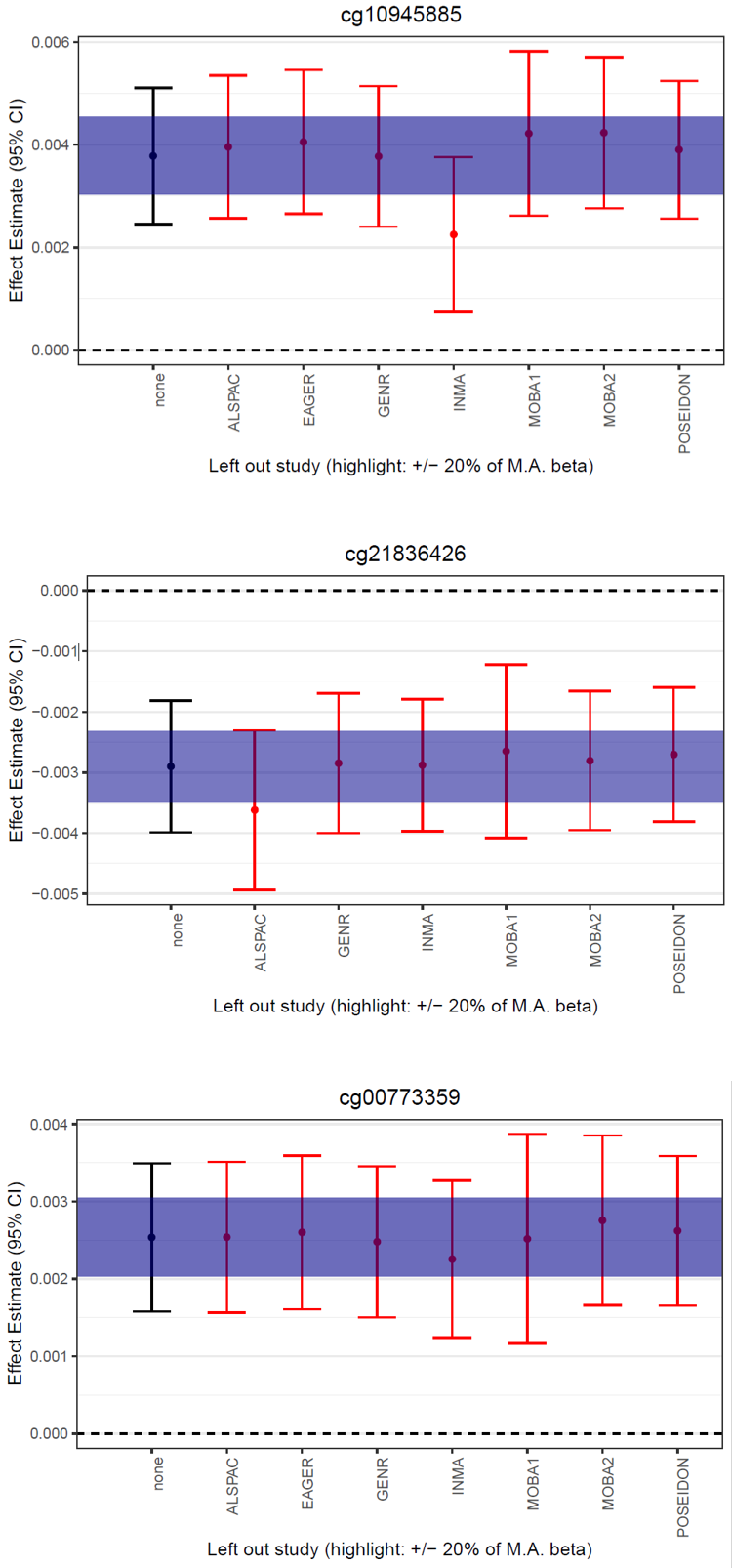


**Supplementary Figure 4 -** Leave-one-out meta-analyses plots for significant CpGs


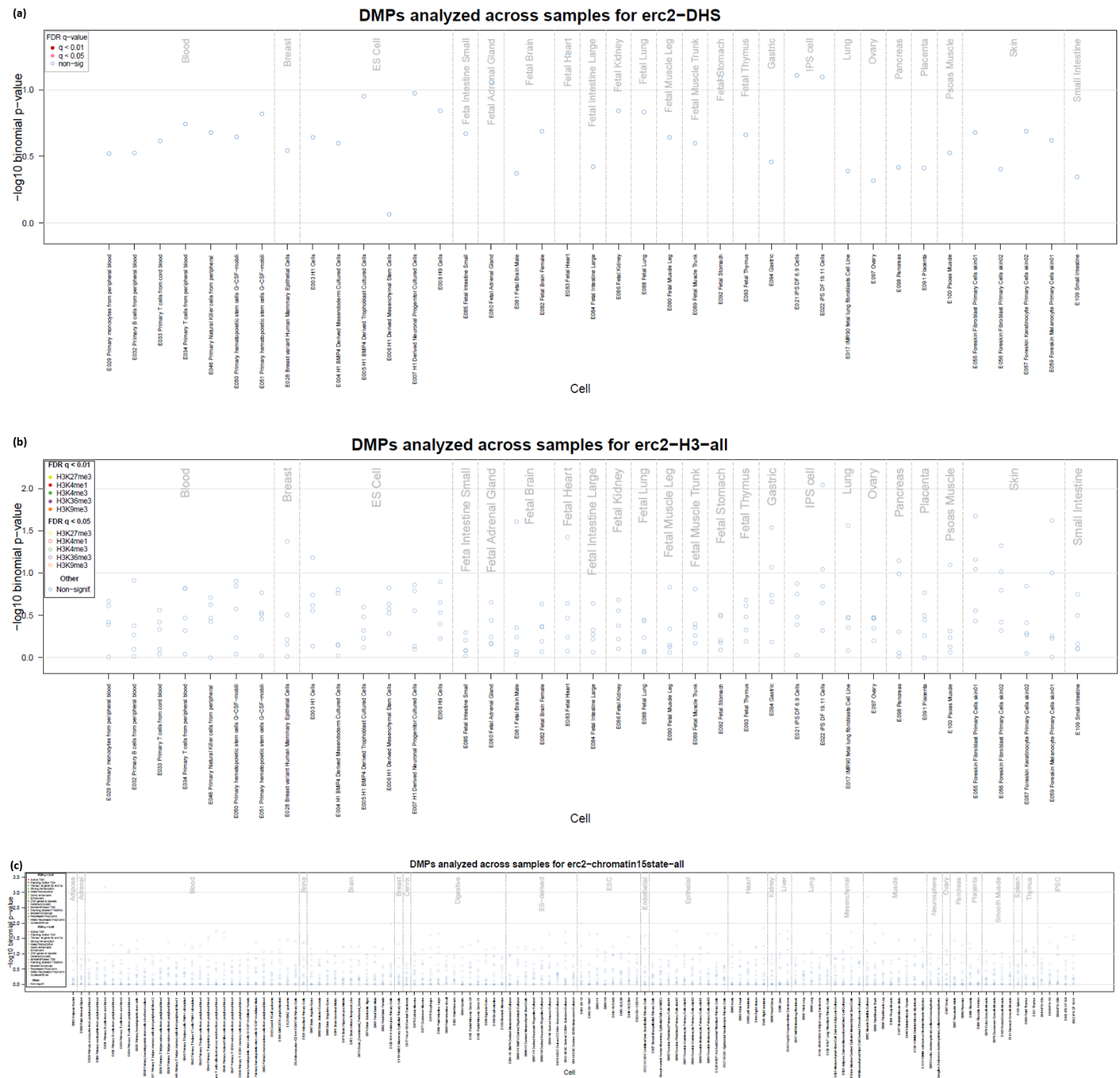


**Supplementary Figure 5 -** Lookup in eForge 2.0 for regulatory elements enrichment using the 118 CpGs

with P < 1×10^−4^ from the main model

(a): DNase1 hotspots

(b): histone marks

(c): chromatin states


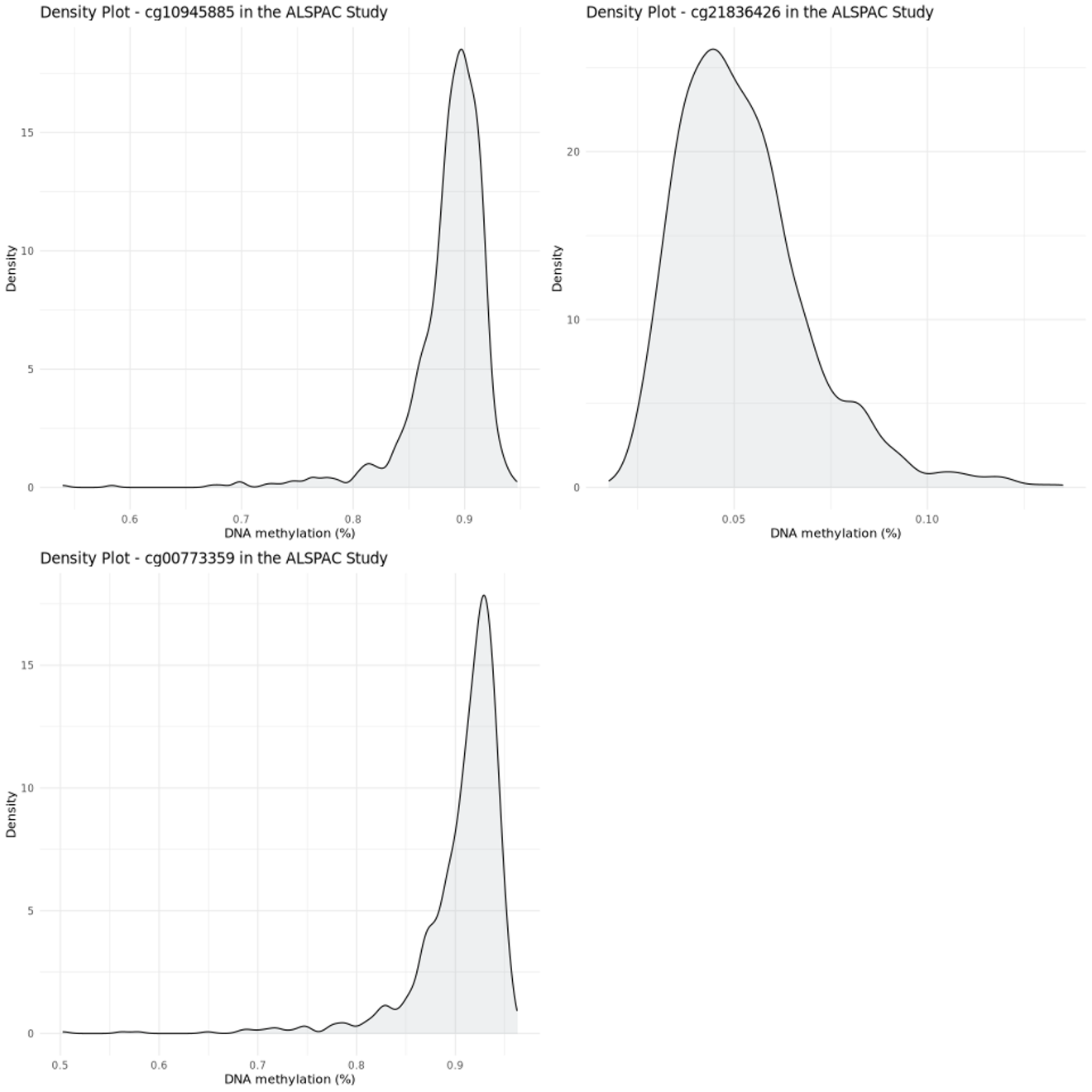


**Supplementary Figure 6** - DNA methylation distribution of the FDR-significant CpGs in the ALSPAC Study


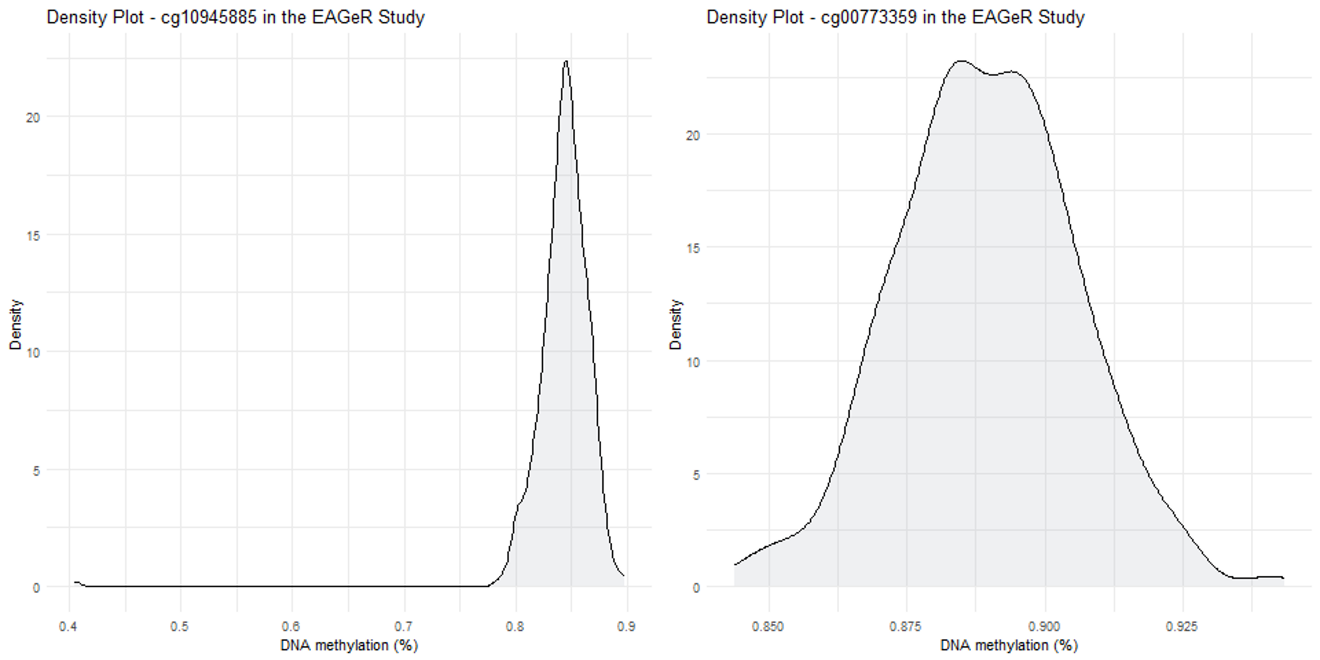


**Supplementary Figure 7 -** DNA methylation distribution of the FDR-significant CpGs in the EAGeR Study


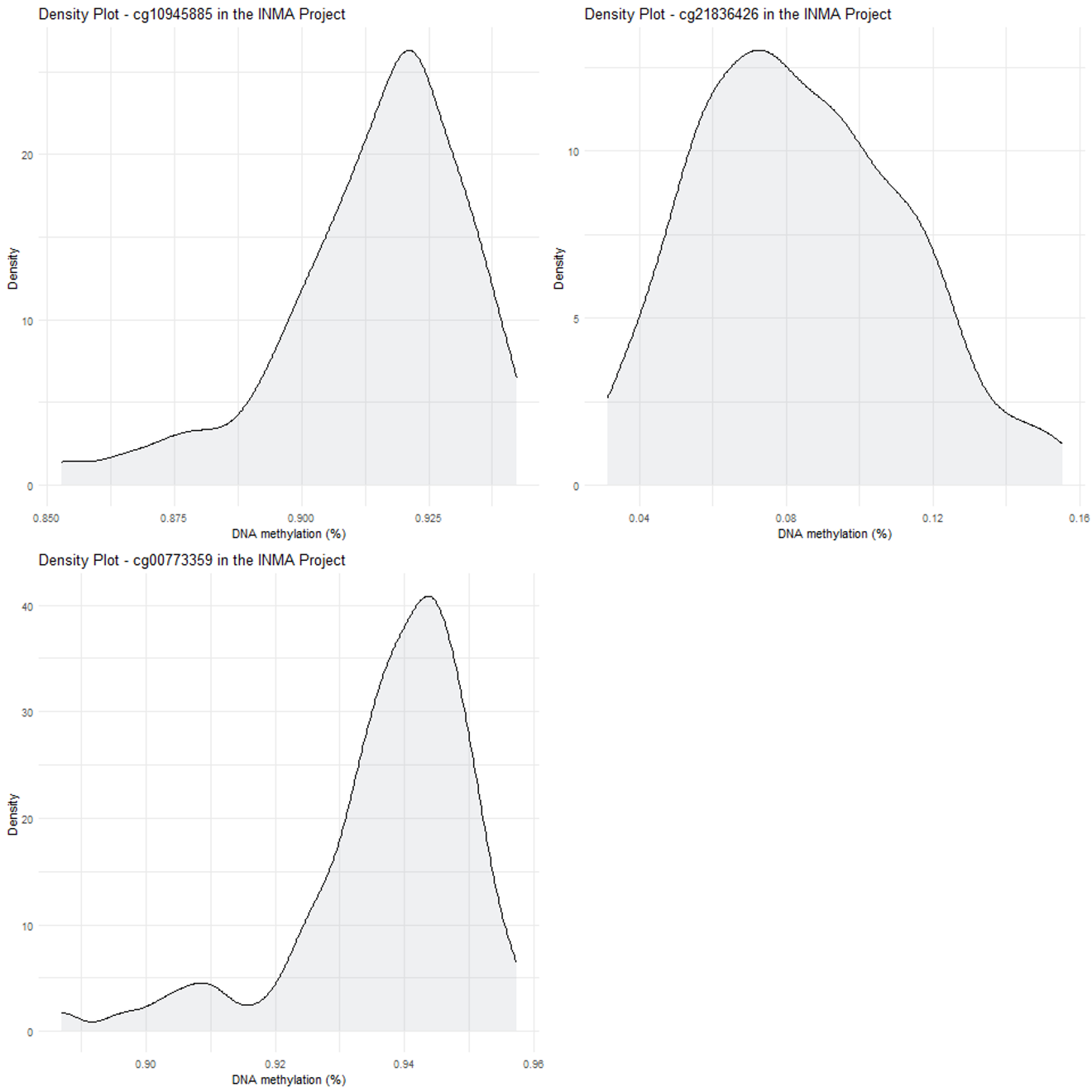


**Supplementary Figure 8 -** DNA methylation distribution of the FDR-significant CpGs in the INMA Project


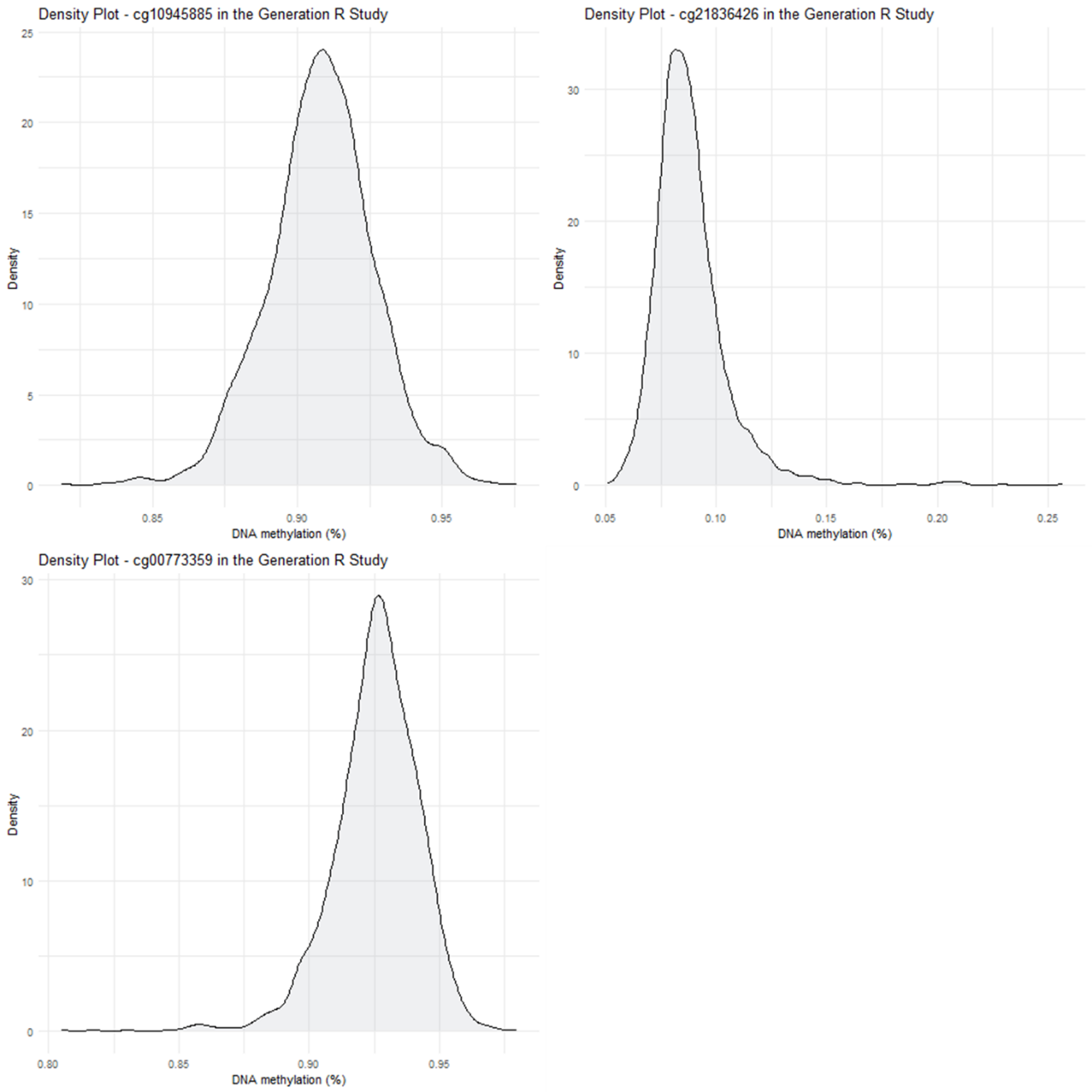


**Supplementary Figure 9 -** DNA methylation distribution of the FDR-significant CpGs in the Generation R Study


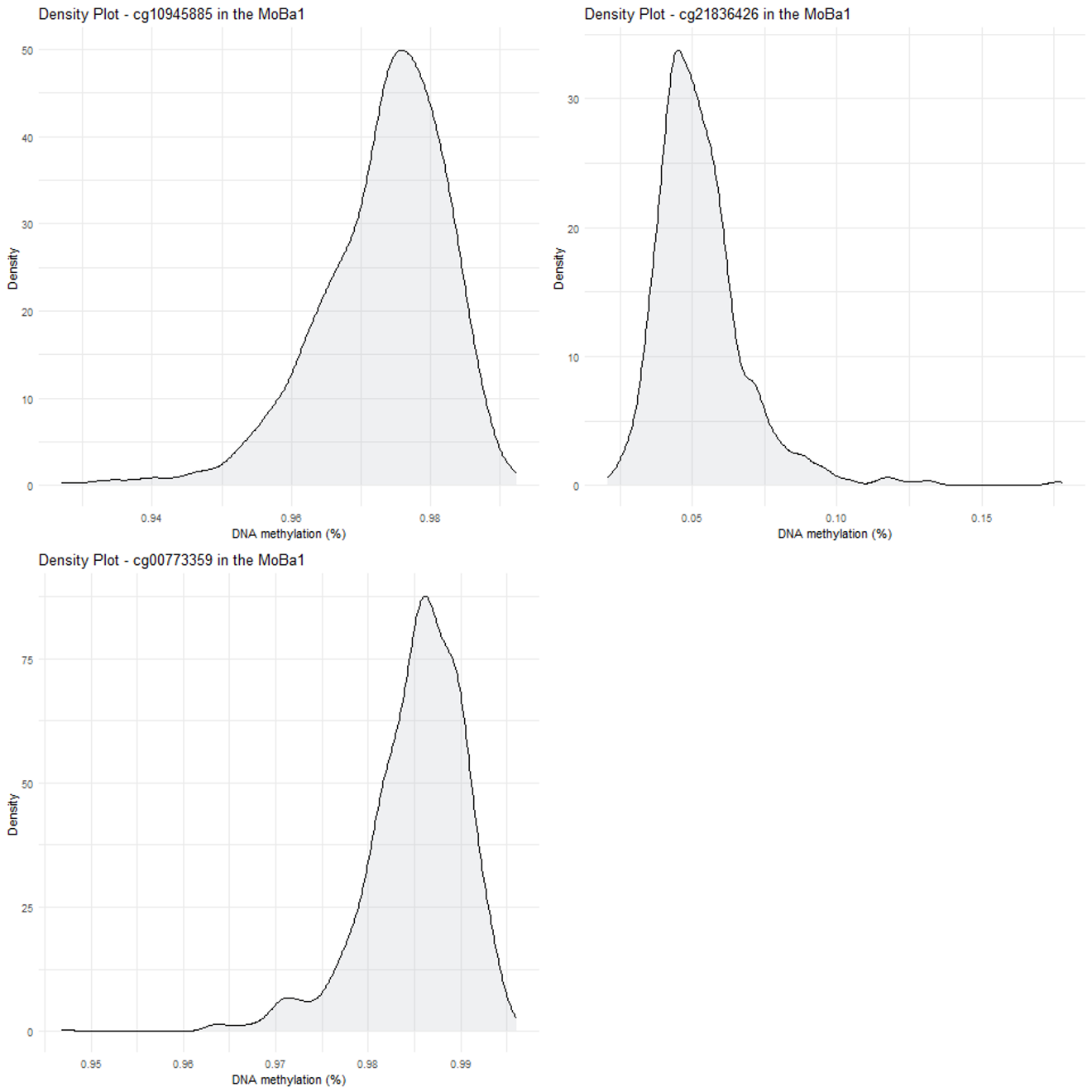


**Supplementary Figure 10** - DNA methylation distribution of the FDR-significant CpGs in MoBa1


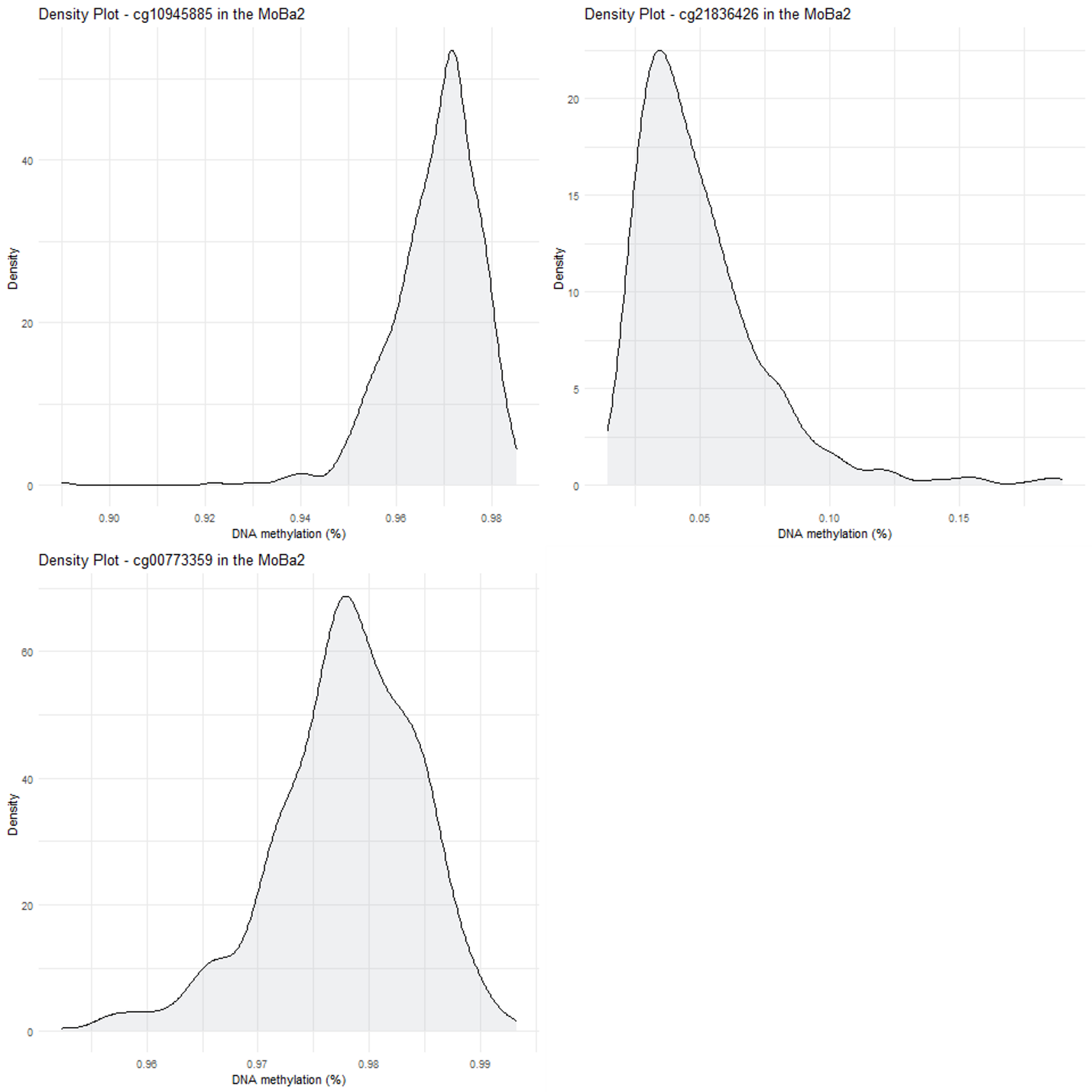


**Supplementary Figure 11** - DNA methylation distribution of the FDR-significant CpGs in MoBa2


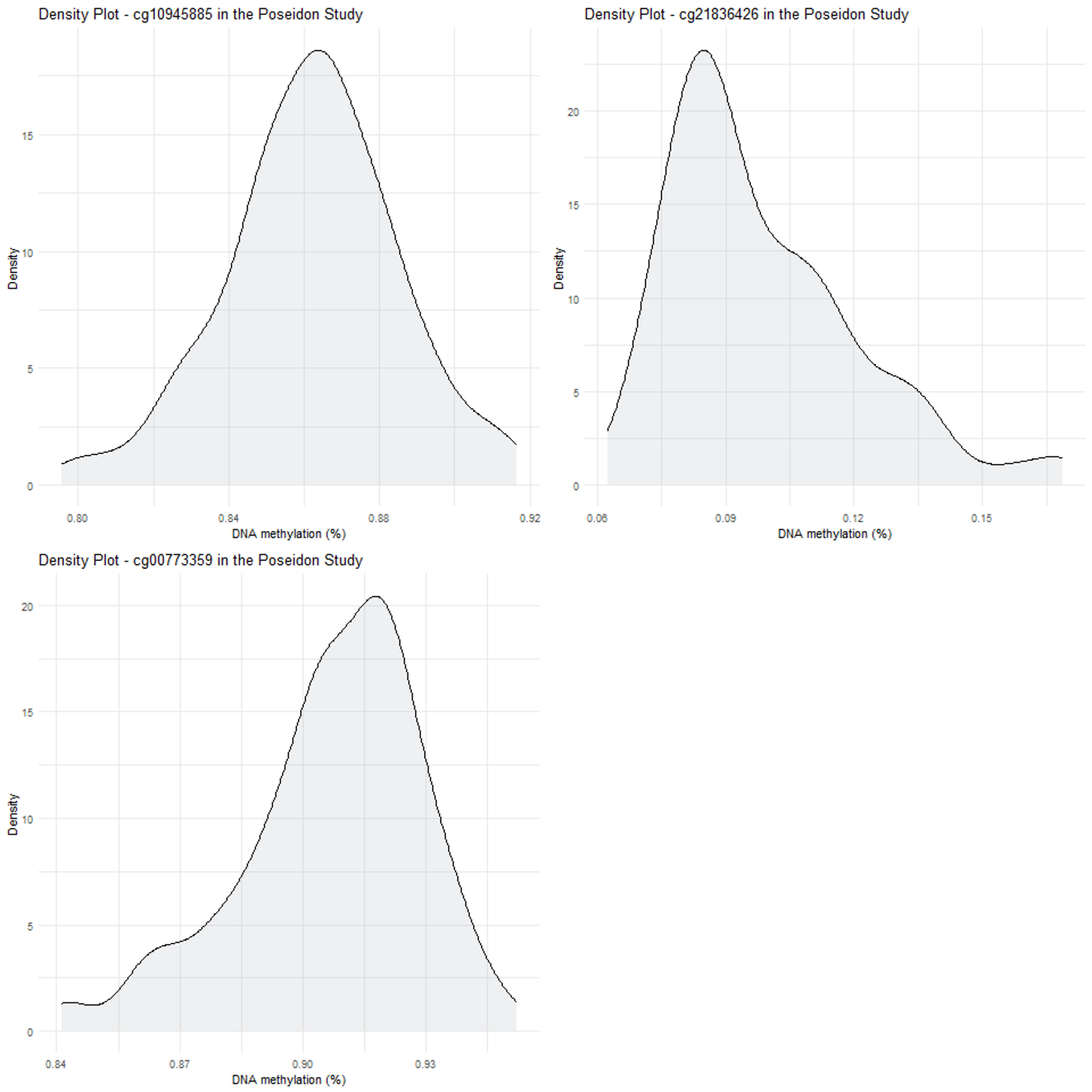


**Supplementary Figure 12** - DNA methylation distribution of the FDR-significant CpGs in POSEIDON
